# The global spectrum of plant form and function: enhanced species-level trait dataset

**DOI:** 10.1038/s41597-022-01774-9

**Published:** 2022-12-07

**Authors:** Sandra Díaz, Jens Kattge, Johannes H. C. Cornelissen, Ian J. Wright, Sandra Lavorel, Stéphane Dray, Björn Reu, Michael Kleyer, Christian Wirth, I. Colin Prentice, Eric Garnier, Gerhard Bönisch, Mark Westoby, Hendrik Poorter, Peter B. Reich, Angela T. Moles, John Dickie, Amy E. Zanne, Jérôme Chave, S. Joseph Wright, Serge N. Sheremetiev, Hervé Jactel, Christopher Baraloto, Bruno E. L. Cerabolini, Simon Pierce, Bill Shipley, Fernando Casanoves, Julia S. Joswig, Angela Günther, Valeria Falczuk, Nadja Rüger, Miguel D. Mahecha, Lucas D. Gorné, Bernard Amiaud, Owen K. Atkin, Michael Bahn, Dennis Baldocchi, Michael Beckmann, Benjamin Blonder, William Bond, Ben Bond-Lamberty, Kerry Brown, Sabina Burrascano, Chaeho Byun, Giandiego Campetella, Jeannine Cavender-Bares, F. Stuart Chapin, Brendan Choat, David Anthony Coomes, William K. Cornwell, Joseph Craine, Dylan Craven, Matteo Dainese, Alessandro Carioca de Araujo, Franciska T. de Vries, Tomas Ferreira Domingues, Brian J. Enquist, Jaime Fagúndez, Jingyun Fang, Fernando Fernández-Méndez, Maria T. Fernandez-Piedade, Henry Ford, Estelle Forey, Gregoire T. Freschet, Sophie Gachet, Rachael Gallagher, Walton Green, Greg R. Guerin, Alvaro G. Gutiérrez, Sandy P. Harrison, Wesley Neil Hattingh, Tianhua He, Thomas Hickler, Steven I. Higgins, Pedro Higuchi, Jugo Ilic, Robert B. Jackson, Adel Jalili, Steven Jansen, Fumito Koike, Christian König, Nathan Kraft, Koen Kramer, Holger Kreft, Ingolf Kühn, Hiroko Kurokawa, Eric G. Lamb, Daniel C. Laughlin, Michelle Leishman, Simon Lewis, Frédérique Louault, Ana C. M. Malhado, Peter Manning, Patrick Meir, Maurizio Mencuccini, Julie Messier, Regis Miller, Vanessa Minden, Jane Molofsky, Rebecca Montgomery, Gabriel Montserrat-Martí, Marco Moretti, Sandra Müller, Ülo Niinemets, Romà Ogaya, Kinga Öllerer, Vladimir Onipchenko, Yusuke Onoda, Wim A. Ozinga, Juli G. Pausas, Begoña Peco, Josep Penuelas, Valério D. Pillar, Clara Pladevall, Christine Römermann, Lawren Sack, Norma Salinas, Brody Sandel, Jordi Sardans, Brandon Schamp, Michael Scherer-Lorenzen, Ernst-Detlef Schulze, Fritz Schweingruber, Satomi Shiodera, Ênio Sosinski, Nadejda Soudzilovskaia, Marko J. Spasojevic, Emily Swaine, Nathan Swenson, Susanne Tautenhahn, Ken Thompson, Alexia Totte, Rocío Urrutia-Jalabert, Fernando Valladares, Peter van Bodegom, François Vasseur, Kris Verheyen, Denis Vile, Cyrille Violle, Betsy von Holle, Patrick Weigelt, Evan Weiher, Michael C. Wiemann, Mathew Williams, Justin Wright, Gerhard Zotz

**Affiliations:** 1 Consejo Nacional de investigaciones Científicas y Técnicas, Instituto Multidisciplinario de Biología Vegetal (IMBIV), Córdoba, Argentina; 2 Facultad de Ciencias Exactas, Físicas y Naturales, Universidad Nacional de Córdoba, Casilla de Correo 495, 5000 Córdoba, Argentina; 3 Max Planck Institute for Biogeochemistry, Hans-Knöll Str. 10, 07745 Jena, Germany; 4 German Centre for Integrative Biodiversity Research (iDiv) Halle-Jena-Leipzig, Leipzig, Germany; 5 A-LIFE, section Systems Ecology, Vrije Universiteit, Amsterdam, The Netherlands; 6 Hawkesbury Institute for the Environment, Western Sydney University, Locked Bag 1797, Penrith, NSW 2751 Australia; 7 School of Natural Sciences, Macquarie University, Sydney, NSW 2109 Australia; 8 Univ. Grenoble Alpes, CNRS, Univ. Savoie Mont Blanc, LECA, Grenoble, France; 9 Univ Lyon, Université Claude Bernard Lyon 1, CNRS, Laboratoire de Biométrie et Biologie Evolutive, F-69100 Villeurbanne, France; 10 Escuela de Biología, Universidad Industrial de Santander, Cra. 27 Calle 9, 680002 Bucaramanga, Colombia; 11 Landscape Ecology Group, Inst. of Biology and Environmental Sciences, University of Oldenburg, 26111 Oldenburg, Germany; 12 University of Leipzig, Leipzig, Germany; 13 Georgina Mace Centre for the Living Planet, Department of Life Sciences, Imperial College London, Silwood Park Campus, Buckhurst Road, Ascot, SL5 7PY UK; 14 Ministry of Education Key Laboratory for Earth System Modelling, Department of Earth System Science, Tsinghua University, Beijing, 100084 China; 15 CEFE, Univ Montpellier, CNRS, EPHE, IRD, Montpellier, France; 16 Plant Sciences (IBG2), Forschungszentrum Jülich, Jülich, Germany; 17 Department of Forest Resources, 1530 Cleveland Ave. N., University of Minnesota, St. Paul, MN 55108 USA; 18 Institute for Global Change Biology and School for Environment and Sustainability, University of Michigan, Ann Arbor, USA; 19 Evolution & Ecology Research Centre, School of Biological, Earth and Environmental Sciences, UNSW, Sydney, NSW 2052 Australia; 20 Wellcome Trust Millennium Building, Royal Botanic Gardens Kew, Wakehurst Place, Ardingly, West Sussex RH17 6TN UK; 21 Department of Biology, University of Miami, Miami, FL 33146 USA; 22 Department of Biological Sciences, George Washington University, Washington, DC 20052 USA; 23 Laboratoire Evolution et Diversité Biologique UMR 5174 CNRS, IRD, Université Paul Sabatier, 31062 Toulouse, France; 24 Smithsonian Tropical Research Institute, Panamá, Panama; 25 Komarov Botanical Institute RAS, Saint Petersburg, Russia; 26 INRAE, University of Bordeaux, BIOGECO, F-33610 Cestas, France; 27 International Center for Tropical Botany, Department of Biological Sciences, Florida International University, Miami, FL 33199 USA; 28 Department of Biotechnologies and Life Sciences (DBSV), University of Insubria, via Dunant 3, IT-21100 Varese, Italy; 29 Department of Agricultural and Environmental Sciences (DiSAA), University of Milan, via Celoria 2, Milan, IT-20133 Italy; 30 Université de Sherbrooke, Sherbrooke, QC Canada; 31 CATIE-Centro Agronómico Tropical de Investigación y Enseñanza, Turrialba, Costa Rica; 32 University of Zurich Department of Geography, Winterthurerstrasse 190, 8057 Zürich, Switzerland; 33 Department of Economics, University of Leipzig, Leipzig, Germany; 34 Remote Sensing Centre for Earth System Research, Leipzig University, Leipzig, Germany; 35 Université de Lorraine, Lorraine, France; 36 Division of Plant Sciences, Research School of Biology, Australian National University, Canberra, 2601 ACT Australia; 37 Department of Ecology, University of Innsbruck, Innsbruck, Austria; 38 University of California, Berkeley, USA; 39 Helmholtz Centre for Environmental Research - UFZ, Department of Computational Landscape Ecology, 04318 Leipzig, Germany; 40 Department of Environmental Science, Policy, and Management, University of California at Berkeley, Berkeley, California USA; 41 Environmental Change Institute, School of Geography and the Environment, University of Oxford, South Parks Road, Oxford, United Kingdom; 42 University of CapeTown, Biological Sciences, Rondebosch, 7701 Cape Town, South Africa; 43 South African Environmental Observation Network, c/o Fynbos node, Private Bag X7, Claremont, 7735 South Africa; 44 Pacific Northwest National Laboratory, Joint Global Change Research Institute at the University of Maryland–College Park, College Park, MD 20740 USA; 45 Kingston University London, Department of Geography, Geology and the Environment, Kingston Upon Thames, UK; 46 Department of Environmental Biology, Sapienza University of Rome, Roma, Italy; 47 Department of Biological Sciences and Biotechnology, Andong National University, Andong, 36729 Korea; 48 School of Biosciences & Veterinary Medicine - Plant Diversity and Ecosystems Management Unit, University of Camerino, Camerino, Italy; 49 Department of Ecology, Evolution and Behavior, University of Minnesota, Saint Paul, MN 55108 USA; 50 Institute of Arctic Biology, University of Alaska Fairbanks, Fairbanks, AK 99775 USA; 51 Department of Plant Sciences, University of Cambridge Conservation Initiative, Pembroke St, Cambridge, CB2 UK; 52 Jonah Ventures, Boulder, CO 80301 USA; 53 Centro de Modelación y Monitoreo de Ecosistemas, Facultad de Ciencias, Universidad Mayor, Santiago, Chile; 54 Institute for Alpine Environment, Eurac Research, Viale Druso 1, 39100 Bozen/Bolzano, Italy; 55 Empresa Brasileira de Pesquisa Agropecuária (EMBRAPA), Belém, PA CEP 66095-100 Brazil; 56 Institute for Biodiversity and Ecosystem Dynamics, University of Amsterdam, Amsterdam, Netherlands; 57 Faculdade de Filosofia, Ciências e Letras - Universidade de São Paulo - Depto. de Biologia, Ribeirão Preto, Brazil; 58 Department of Ecology and Evolutionary Biology, University of Arizona, Tucson, AZ 85721 USA; 59 The Santa Fe Institute 1399 Hyde Park Rd., Santa Fe, NM 87501 USA; 60 Biology department, BioCost group, Centro interdisciplinar de química y biología CICA, University of A Coruña, 15071A Coruña, Spain; 61 Institute of Ecology, College of Urban and Environmental Sciences, and Key Laboratory for Earth Surface Processes of the Ministry of Education, Peking University, Beijing, 100871 China; 62 Grupo de Investigación en Biodiversidad y Dinámica de Ecosistémas Tropicales - Universidad del Tolima, Ibagué, Colombia; 63 Centro Forestal Tropical Bajo Calima, Universidad del Tolima, Buenaventura, Costa Rica; 64 Instituto Nacional de Pesquisas da Amazônia, Av. André Araújo, 2.936 - Petrópolis - CEP, 69067-375 Manaus, AM Brasil; 65 Ecological Flora of the British Isles, Bath, UK; 66 Université de Rouen Normandie- INRAE, 76000 Rouen, France; 67 Theoretical and Experimental Ecology Station, CNRS, Paul Sabatier University, 09200 Moulis, France; 68 IMBE, Aix Marseille Univ, CNRS, IRD, Avignon Univ, Marseille, France; 69 Department of Organismic and Evolutionary Biology, Harvard University, Cambridge, MA 02138 USA; 70 School of Biological Sciences, The University of Adelaide, Adelaide, SA 5005 Australia; 71 Departamento de Ciencias Ambientales y Recursos Naturales Renovables, Facultad de Ciencias Agronómicas, Universidad de Chile, Av. Santa Rosa 11315, La Pintana, 8820808 Santiago, Chile; 72 Institute of Ecology and Biodiversity (IEB), Concepción, Chile; 73 University of Reading, Reading, UK; 74 Nova Pioneer, Global Systems and Analytics, Paulshof, Gauteng, South Africa; 75 School of Molecular and Life Sciences, Curtin University, Perth, WA Australia; 76 College of Science, Health, Engineering and Education, Murdoch University, Murdoch, WA Australia; 77 Senckenberg Biodiversity and Climate Research Centre (SBiK-F), Senckenberganalge 25, 60325 Frankfurt/Main, Germany; 78 Institut of Physcial Geography, Goethe University, Altenhöferallee 1, 60438 Frankfurt/Main, Germany; 79 Plant Ecology, University of Bayreuth, 95447 Bayreuth, Germany; 80 Santa Catarina State University, Lages, SC Brazil; 81 University of Melbourne, Melburne, Australia; 82 CSIRO, Melburne, Canada; 83 Department of Earth System Science, Woods Institute for the Environment, and Precourt Institute for Energy, Stanford, CA USA; 84 Research Institute of Forests and Rangeland, Agricultural Research, Education and Extension Organization (AREEO), Tehran, Iran; 85 Institue of Systematic Botany and Ecology, Ulm University, Albert-Einstein-Allee 11, 89081 Ulm, Germany; 86 Graduate School of Environment and Information Sciences, Yokohama National University, Yokohama, 240-8501 Japan; 87 Institute for Biochemistry and Biology, University of Potsdam, Potsdam, Germany; 88 Biodiversity, Macroecology & Biogeography, University of Goettingen, Büsgenweg 1, D-37077 Göttingen, Germany; 89 Department of Ecology and Evolutionary Biology, University of California, Los Angeles, 621 Charles E. Young Drive South, Los Angeles, California 90095 USA; 90 Wageningen University, Droevendaalsesteeg 4, 6700AA Wageningen, Netherlands; 91 Land Life Company, Mauritskade 63, 1092AD Amsterdam, Netherlands; 92 Centre of Biodiversity and Sustainable Land Use (CBL), University of Goettingen, Büsgenweg 1, D-37077 Göttingen, Germany; 93 Helmholtz Centre for Environmental Research - UFZ, Dept. Community Ecology, Theodor-Lieser-Str. 4, 06120 Halle, Germany; 94 Martin Luther University Halle-Wittenberg, Geobotany and Botanical Garden, Am Kirchtor 1, 06108 Halle, Germany; 95 Forestry and Forest Products Research Institute, Tsukuba, 305-8687 Japan; 96 Department of Plant Sciences, University of Saskatchewan, Saskatoon, SK Canada; 97 Department of Botany, University of Wyoming, 1000 East University Ave, Laramie, WY 82071 USA; 98 School of Geography, University of Leeds, Leeds, UK; 99 Université Clermont Auvergne, INRAE, VetAgro Sup, UMR Ecosystème Prairial, 63000 Clermont Ferrand, France; 100 Instituto de Ciências Biológicas e da Saúde, Universidade Federal de Alagoas, Maceió, AL 57072-900 Brazil; 101 Department of Biological Sciences, University of Bergen, Bergen, Norway; 102 School of Geosciences, University of Edinburgh, Edinburgh, EH93FF United Kingdom; 103 CREAF, Cerdanyola del Vallès, 08193 Catalonia, Spain; 104 ICREA, Pg. Lluís Companys 23, 08010 Barcelona, Spain; 105 Department of Biology, University of Waterloo, Waterloo, ON N2L 3G1 Canada; 106 Forest Products Lab, USDA Forest Service, Madison, Wisconsin USA; 107 Department of Biology, Vrije Universiteit Brussel, Pleinlaan 2, 1050 Brussels, Belgium; 108 Department of Plant Biology, University of Vermont, Burlington, Vermont 05405 USA; 109 Instituto Pirenaico de Ecología (IPE-CSIC), Av. Montañana, 1005, 50059 Zaragoza, Spain; 110 Biodiverstiy and Conservation Biology, Swiss Federal Research Institute WSL, Birmensdorf, 8903 Switzerland; 111 Geobotany, Faculty of Biology, University of Freiburg, Schänzlestr. 1, 79104 Freiburg, Germany; 112 Estonian University of Life Sciences, Kreutzwaldi 1, Tartu, 51006 Estonia; 113 Estonian Academy of Sciences, Kohtu 6, Tallinn, 10130 Estonia; 114 CSIC, Global Ecology Unit CREAF-CSIC-UAB, Bellaterra, 08193 Catalonia Spain; 115 Institute of Biology Bucharest, Romanian Academy, 060031 Bucharest, Romania; 116 Institute of Ecology and Botany, Centre for Ecological Research, 2163 Vácrátót, Hungary; 117 Department of Ecology and Plant Geography, Faculty of Biology, Moscow Lomonosov State University, 119234 Moscow, Russia; 118 Graduate School of Agriculture, Kyoto University, Kyoto, 606-8502 Japan; 119 Wageningen Environmental Research, PO Box 47, NL-6700 AA Wageningen, Wageningen, Netherlands; 120 Centro de Investigaciones sobre Desertificación, Consejo Superior de Investigaciones Científicas (CIDE-CSIC), Valencia, 46113 Spain; 121 Centro de Investigacion en Biodiversidad y Cambio Global (CIBC-UAM), Departamento de Ecología, Universidad Autónoma de Madrid, E-28049 Madrid, Spain; 122 Department of Ecology, Universidade Federal do Rio Grande do Sul, Porto Alegre, RS 91501-970 Brazil; 123 Snow and Mountain Research Center of Andorra, Andorran Research + Innovation, Avda. Rocafort 21-23 Sant Julià de Lòria, Principality of Andorra, Julià de Lòria, Andorra; 124 Institute of Ecology and Evolution, Friedrich-Schiller University Jena, Philosophenweg 16, D-07743 Jena, Germany; 125 Instituto de Ciencias de la Naturaleza, Territorio y Energías Renovables, Pontificia Universidad Católica del Perú, Lima, Peru; 126 Department of Biology, Santa Clara University, 500 El Camino Real, Santa Clara, CA 95053 USA; 127 Department of Biology, Algoma University, Sault Ste, Marie, Ontario P6A 6J8 Canada; 128 Landscape Dynamics, Swiss Federal Research Institute WSL, Birmensdorf, 8903 Switzerland; 129 Department of Global Liberal Studies, Faculty of Global Liberal Studies, Nanzan University, Aichi, 466-8673 Japan; 130 Center for Southeast Asian Studies (CSEAS), Kyoto University, 46 Shimoadachi-cho, Yoshida, Sakyo-ku, Kyoto, 606-8501 Japan; 131 Embrapa Clima Temperado, Pelotas, RS Brazil; 132 Centre for Environmental Sciences, Hasselt University, Hasselt, Belgium; 133 Institute of Environmental Sciences, Leiden University, Einsteinweg 2, 2333 CC Leiden, Netherlands; 134 Department of Evolution, Ecology, and Organismal Biology, University of California Riverside, Riverside, California 92521 USA; 135 School of Biological Sciences, University of Aberdeen, Aberdeen, AB24 3FX UK; 136 Department of Biological Sciences, University of Notre Dame, Notre Dame, IN 46556 USA; 137 Department of Animal and Plant Sciences, University of Sheffield, Western Bank, Sheffield, S10 2TN UK; 138 Département de biologie des organismes et écologie, Université libre de Bruxelles, Roosevelt, Belgique; 139 Departamento de Ciencias Naturales y Tecnología, Universidad de Aysén, Coyhaique, Chile; 140 Center for Climate and Resilience Research CR2, Santiago, Chile; 141 National Museum of Natural Sciences, MNCN, CSIC, Serrano 115, 28006 Madrid, Spain; 142 University Rey Juan Carlos, Mostoles, Área de Biodiversidad y Conservación C/Tulipán sn, 28033 Móstoles, Madrid Spain; 143 LEPSE, Univ Montpellier, INRAE, Institut Agro, Montpellier, France; 144 Forest & Nature Lab, Department of Environment, Ghent University, Geraardsbergsesteenweg 267, B-9090 Melle-Gontrode, Belgium; 145 National Science Foundation, Division of Environmental Biology, Alexandria, VA 22314 USA; 146 Department of Biology, University of Wisconsin - Eau Claire, Eau Claire, WI 54702 USA; 147 Department of Biology, Duke University, Durham, NC USA; 148 Functional Ecology Group, Inst. of Biology and Environmental Sciences, University of Oldenburg, 26111 Oldenburg, Germany

**Keywords:** Biodiversity, Biogeography, Macroecology

## Abstract

Here we provide the ‘Global Spectrum of Plant Form and Function Dataset’, containing species mean values for six vascular plant traits. Together, these traits –plant height, stem specific density, leaf area, leaf mass per area, leaf nitrogen content per dry mass, and diaspore (seed or spore) mass – define the primary axes of variation in plant form and function. The dataset is based on ca. 1 million trait records received via the TRY database (representing ca. 2,500 original publications) and additional unpublished data. It provides 92,159 species mean values for the six traits, covering 46,047 species. The data are complemented by higher-level taxonomic classification and six categorical traits (woodiness, growth form, succulence, adaptation to terrestrial or aquatic habitats, nutrition type and leaf type). Data quality management is based on a probabilistic approach combined with comprehensive validation against expert knowledge and external information. Intense data acquisition and thorough quality control produced the largest and, to our knowledge, most accurate compilation of empirically observed vascular plant species mean traits to date.

## Abstract (summary)

**Table:** 

Measurement(s)	plant trait
Technology Type(s)	various
Factor Type(s)	none
Sample Characteristic - Organism	Tracheophyta
Sample Characteristic - Environment	natural environment
Sample Characteristic - Location	global

## Background & Summary

Plant traits are the morphological, chemical, physiological or phenological properties of individuals^1^. They determine how plants as primary producers capture, process and store resources, how they respond to their abiotic and biotic environment and disturbances, and how they affect other trophic levels and the fluxes of water, carbon and energy through ecosystems^2–8^.

Despite the overwhelming diversity of plant forms and life histories on Earth, single plant organs, such as leaves, stems, or seeds, show comparatively few essential trait combinations^9^. Evidence for recurrent trait syndromes beyond the level of single organs has been rare, restricted geographically or taxonomically, and often contradictory. Díaz *et al*.^9^ addressed this question by analyzing the worldwide variation in six major traits critical to growth, survival and reproduction, namely: plant height (H), stem specific density (SSD), leaf area (LA), leaf mass per area (LMA), leaf nitrogen content per dry mass (N_mass_) and diaspore (seed or spore) mass (SM). Díaz *et al*.^9^ found that occupancy of the six-dimensional trait space is highly constrained, and is captured in a two-dimensional global spectrum of plant form and function, indicating strong correlation and trade-offs among traits. These results provide a foundation and baseline for studies of plant evolution, comparative plant and ecosystems ecology, and predictive modelling of future vegetation based on continuous variation in essential plant functional dimensions.

Here we provide the trait dataset that served as basis for the analysis of the global spectrum of plant form and function presented in Díaz *et al*.^9^ –the ‘Global Spectrum of Plant Form and Function Dataset’ (short here ‘Global Spectrum Dataset’). The dataset is predominantly based on trait records compiled in the TRY database^10,11^ and provides trait values corresponding –to the extent possible–to mature and healthy plants grown under natural conditions within the species distribution range. The dataset provides species mean values for the six plant traits mentioned above plus leaf dry matter content, used for the imputation of stem specific density. The dataset covers >46,000 of the approximately 391,000 vascular plant species known to science^12^. Despite the rapid development of large plant trait datasets, the Global Spectrum Dataset stands out in terms of coverage and reliability. First, it provides quantitative information for a very high number of species, including about 5% of them with ‘complete coverage’ (all six traits). Second, it represents a unique combination of probabilistic outlier detection and comprehensive validation of trait values against expert knowledge and external information for data quality assurance. Third, it contains the attribution of data to original references, even if datasets contributed to TRY had been assembled from multiple original sources.

The quantitative trait data are enhanced by higher-level taxonomic information, based on the Angiosperm Phylogeny APG III (http://www.mobot.org/MOBOT/research/APweb/) and categorical traits, based on the ‘TRY – Categorical Traits Dataset’^13^, enriched by field data and various literature sources. This information facilitates stratification of species and quantitative traits according to phylogenetic and morpho-functional criteria.

The present dataset results from the integration of trait measurements from many datasets received via TRY and additional, partly unpublished, data. The data come from largely independent studies, that address a wide variety of questions at different scales, and using different measurement methods, units and terminologies^14^. The development of the dataset therefore faced three challenges: (1) to derive a dataset of species mean values covering all six traits with the aim of being representative of vascular plant species worldwide; (2) to detect erroneous trait records (due to errors in sampling, measurement, unit conversion, etc.); and (3) to ensure that correctly measured extreme values of traits in nature were not mistakenly identified as outliers and therefore excluded from the dataset. To deal with these challenges, we collected as many trait observations as possible. The dataset was developed over a period of six years (2009–2015) with continuous addition of new trait records as data became available. The final dataset is based on almost 1 million trait records, which can be traced back to ca. 2,500 references (see file: ‘References_original_sources.xlsx’). We identified outliers and potential errors based on a probabilistic approach^10^ combined with validation by domain experts and external information.

These combined efforts of data acquisition, integration and quality control resulted in the most comprehensive and probably most accurate dataset for species mean traits of vascular plants published so far.

## Methods

### Selection of plant traits

There is an extensive literature summarized in Díaz *et al*.^9^ and Pérez-Harguindeguy *et al*.^6^ supporting the key importance of the six core traits chosen – H, SSD, LA, LMA, N_mass_ and SM – to growth, survival and reproduction. Díaz *et al*.^9^ went further by showing that, together, these traits capture the essence of plant form and function at the broad scale: a two-dimensional space, with one major dimension reflecting the size of whole plants and its organs, and the other representing a balance between leaf construction cost against growth potential, captures roughly three-quarters of total trait variation. The core quantitative traits were complemented with the categorical traits: woodiness, growth form, succulence, adaptation to terrestrial or aquatic habitats, nutrition type, and leaf type.

### Definition of traits

In the following section we provide the names and definitions used for the continuous traits in the original publication of the global spectrum^9^, plus the names and definitions used in the Thesaurus Of Plant Characteristics (TOP)^14^. The detailed rationale, ecological meaning and key references for each of them can be found in the methods section of Díaz *et al*.^9^ and in Garnier *et al*.^7^. For the categorical traits we provide names, definition where available, and the categories used in the database. Traits were mostly measured following the protocols and definitions specified in the ‘New Handbook for Standardised Measurement of Plant Functional Traits Worldwide’^6^ (http://www.nucleodiversus.org). In the case of data from the LEDA database, measurements followed the protocols developed in the context of the LEDA project^16^ (https://www.leda-traitbase.org). In the case of published datasets individual measurement protocols are available in the original publications listed in Table S1.

#### Plant height (H) (unit: m)

Adult plant height, i.e. typical height of the upper boundary of the main photosynthetic tissues at maturity (TOP: vegetative plant height; the plant height considering the highest vegetative component).

#### Stem specific density (SSD) (unit: mg mm^−3^)

Stem dry mass per unit of stem fresh volume (TOP: stem specific density; the ratio of the mass of the stem or a unit thereof assessed after drying to its volume assessed without drying). SSD is much more commonly measured on woody species (particularly trees), than on non-woody species. Therefore, gaps in SSD for non-woody species were filled by estimates derived from leaf dry matter content (see Data Imputation below).

#### Leaf area (LA) (unit: mm^2^)

One-sided surface area of an individual lamina (TOP: leaf lamina area; the area of the leaf lamina in the one-sided projection; in case of compound leaves the area of a leaflet lamina).

#### Leaf mass per area (LMA) (unit: g m^−2^)

Leaf dry mass per unit of lamina surface area (TOP: leaf mass per area, the ratio of the dry mass of a leaf to its area).

#### Leaf nitrogen per mass (N_mass_) (unit: mg g^−1^)

Leaf nitrogen content per unit of lamina dry mass (leaf total N) (TOP: leaf nitrogen content per leaf dry mass; the ratio of the quantity of nitrogen in the leaf or component thereof, i.e. leaf lamina or leaflet, per respective unit dry mass).

#### Diaspore mass (SM) (unit: mg)

Dry mass of an individual seed or spore plus any additional structures that assist dispersal and do not easily detach (TOP: seed dry mass**;** mass of an individual seed or spore assessed after drying; seed dry mass). Spore mass of pteridophytes, rarely reported in the literature, was estimated from published values of diaspore diameter and density (see Data Imputation below).

#### Leaf dry matter content (LDMC) (unit: g g^−1^)

The ratio of the dry mass of the leaf or component thereof, i.e. leaf lamina, to the corresponding water saturated fresh mass. In addition to the six focal traits, we compiled LDMC for herbaceous plants to calculate missing values for SSD (see Data Imputation below).

#### Adaptation to terrestrial or aquatic habitats

On the basis of the type of habitat in which the species naturally grows. Categories: aquatic, aquatic/semiaquatic, semiaquatic, terrestrial.

#### Woodiness

A feature of the whole plant defining the occurrence and distribution of wood along the stem. Categories: woody, non-woody, semi-woody (woody at base of stem(s) only).

#### Growth form

Growth form is mainly determined by woodiness and the direction and extent of growth, and any branching of the main shoot axis or axes. Categories: bamboo graminoid, climber, fern, herbaceous graminoid, herbaceous non-graminoid, herbaceous non-graminoid/shrub, succulent, shrub, shrub/tree, tree, other.

#### Succulence

Succulence characterizes plants with parts that are thickened, fleshy, and engorged, usually to retain water in conditions where climate or soil characteristics strongly limit water availability to plants. This criterion aims to provide more detailed information to the succulent growth form whenever available. Categories: leaf and stem succulent, leaf rosette and stem succulent, leaf rosette succulent, leaf rosette succulent (tall), leaf succulent, stem succulent, stem succulent (short), stem succulent (tall), succulent.

#### Nutrition type

Nutrition type here refers to whether the major source of energy and nutrients for the plant is photosynthesis, animals, dead material or other plants. Parasitism categories: hemiparasitic, holoparasitic, independent, parasitic. Carnivory categories: carnivorous, detritivorous.

According to the ‘New Handbook for Standardised Measurement of Plant Functional Traits Worldwide’^6^ succulence and nutrition type are part of growth form. We here treat them separately for simplicity and to avoid combined categories.

#### Leaf type

A classification of presence/absence of photosynthetic active leaves and their basic forms. Categories: broadleaved, needleleaved, scale-shaped, scale-shaped/needleleaved, photosynthetic stem.

### Definition of representative trait records

The six core quantitative traits certainly show intraspecific variation, amongst others caused by different ontogenetic stages and growth conditions. The dataset, focused on mean trait values for species rather than intraspecific variation, was intended to represent species mean trait values for mature and healthy (not obviously unhealthy) plants grown under natural conditions within the species distribution range. Leaf traits were intended to represent young but fully expanded and healthy leaves from the light exposed top canopy. Trait records not conforming to these requirements, i.e. records from plants grown in laboratories under experimental conditions and records measured on juvenile plants, were excluded from the dataset. This decision was made based on the respective metadata in the TRY database (see below).

### Data sources

The vast majority of quantitative trait data was provided by the TRY Plant Trait Database^10^ (https:// www.try-db.org, TRY version 2.0 accessed July 2010, updated by TRY version 3.0 accessed May 2015). This dataset was supplemented by a small number of published data not included in TRY and original unpublished data contributed by W. J. Bond, J. H. C. Cornelissen, S. Díaz, L. Enrico, M. T. Fernandez-Piedade, L. D. Gorné, D. Kirkup, M. Kleyer, N. Salinas, E.-D. Schulze, K. Thompson, and R. Urrutia-Jalabert.

Categorical traits were derived from the TRY Categorical Traits Dataset (https://www.try-db.org/TryWeb/Data.php#3), enhanced by field data and various literature sources.

The datasets contributing via TRY to the quantitative traits are described in Supplementary Table S1, which contains data from refs. ^4^,^16^–^233^ and the following unpbublished datasets: French Weeds Trait Database; Photosynthesis and Leaf Characteristics Database; South African Woody Plants Database (ZLTP); Tundra Plant Traits Database; Leaf N-Retention Database; Traits for Herbaceous Species from Andorra; Leaf Characteristics of *Pinus sylvestris* and *Picea abies*; Plant Coastal Dune Traits (France, Aquitaine); Dispersal Traits Database; LABDENDRO Brazilian Subtropical Forest Traits Database; Growth and Herbivory of Juvenile Trees; Cold Tolerance, Seed Size and Height of North American Forest Tree Species; Harze Trait Intravar: SLA; LDMC and Plant Height for Calcareous Grassland Species in South Belgium; Functional Traits for Restoration Ecology in the Colombian Amazon; Komati Leaf Trait Data; Baccara - Plant Traits of European Forests; Traits of Bornean Trees Database; Meadow Plant Traits: Biomass Allocation, Rooting depth; New South Wales Plant Traits Database; Traits for Herbaceous Species from Andorra; Catalonian Mediterranean Shrubland Trait Database; The Netherlands Plant Height Database; Plant Traits from Spanish Mediterranean Shrublands; Crown Architecture Database; Maxfield Meadow, Rocky Mountain Biological Laboratory – LMA; Herbaceous Plants Traits From Southern Germany; Leaf Area, Dry Mass and SLA Dataset; Herbaceous Leaf Traits Database Old Field New York; Plant Functional Traits From the Province of Almeria, Spain; Traits for Common Grasses and Herbs in Spain; Midwestern and Southern US Herbaceous Species Trait Database; Overton/Wright New Zealand Database; San Lorenzo Epiphyte Leaf Traits Database.

The reference for each individual trait record contributing via TRY to the Global Spectrum Dataset before exclusion of non-representative trait records, errors and duplicates is documented in the data file ‘References.xlsx’.

### Data integration and quality management

#### Semantic integration of terminologies from different datasets

Ecological studies are carried out for a large number of different questions at different scales and researchers often work independently and with little coordination among them. This results in idiosyncratic datasets using heterogeneous terminologies^14^. The first step was therefore a semantic integration of terminologies. The core traits were standardized according to the definitions and measurement protocols provided in the Thesaurus Of Plant Characteristics (TOP)^14^ and the ‘New Handbook for Standardised Measurement of Plant Functional Traits Worldwide’^6,15^. The metadata for plant and organ maturity (juvenile, mature), health (healthy, not healthy), growth conditions (natural conditions, experimental conditions), and sun- versus shade-grown leaves were harmonized across datasets.

#### Consolidation of taxonomy

Species names were standardized and attributed to families according to The Plant List (http://www.theplantlist.org), the commonly accepted list for vascular plants at the time of publication of Díaz *et al*.^9^, using TNRS^234,235^, complemented by manual standardization by experts. Attribution of families to higher-rank groups was made according to APG III (2009) (http://www.mobot.org/MOBOT/research/APweb/).

#### Conversion and correction of units, and exclusion of errors

Different datasets often used different units for the same trait. After conversion to the standardized unit per trait, differences among datasets - sometimes in the order of magnitude - became obvious. These differences could often be traced back to errors in the original units and were corrected. Obvious errors (e.g. impossible trait values like LMA < 0 g/m^2^) were excluded from the dataset.

#### Data imputation

To improve the number of species with values for all six core traits, trait records for stem SSD, LMA, N_mass_ and SM were complemented by trait values derived from records of related traits:

#### - Imputation of SSD

Trait records for SSD are available for a very large number of woody species, but only for very few herbaceous species. To incorporate this fundamental trait in the analyses by Díaz *et al*.^9^, we complemented SSD of herbaceous species using an estimation based on leaf dry matter content (LDMC), a much more widely available trait, and its close correlation to stem dry matter content (StDMC, the ratio of stem dry mass to stem water-saturated fresh mass). StDMC is a good proxy of SSD in herbaceous plants with a ratio of approximately 1:1^199^, despite substantial differences in stem anatomy among botanical families^236^, including those between non-monocotyledons and monocotyledons (where sheaths were measured). We used a data set of 422 herbaceous species collected in the field across Europe and Israel, and belonging to 31 botanical families, to parameterize linear relationships of StDMC to LDMC. The slopes of the relationship were significantly higher for monocotyledons than for other angiosperms (F = 12.3; P < 0.001, from a covariance analysis); within non-monocotyledons, the slope for Fabaceae was higher than that for species from other families (F = 4.5; P < 0.05, from a covariance analysis). We thus used three different equations to predict SSD for 1963 herbaceous species for which LDMC values were available in TRY (Table 1): one for monocotyledons, one for Fabaceae, and a third one for other non-monocotyledons. Estimated data are flagged.

**Table 1:** Summary statistics for model I regressions between LDMC and StDMC (dependent variable) for the whole data set and various subsets of species.

Dataset	Slope (SE)	Intercept (SE)	r^2^	N
All species	0.698 (0.042)	0.058 (0.011)	0.398***	422
Monocots	*0.888* (0.071)	*0.027* (0.022)	0.467***	181
Fabaceae	*0.692* (0.129)	*0.048* (0.033)	0.367***	52
All dicots except Fabaceae	*0.524* (0.076)	*0*.*096* (0.017)	0.203***	188

Coefficients in italics were used in linear regressions to predict StDMC from LDMC in various subsets of herbaceous species for which LDMC values were available in the TRY database. Units for StDMC and LDMC are g g^−1^, giving SSD estimates in equivalent units of mg mm^−3^. Note that in Díaz *et al*.^9^, the coefficients of the regressions are given for LDMC and StDMC values expressed in mg g^−1^, yielding SSD estimates in g cm^−3^.

#### - Imputation of LMA

Trait records for SLA (leaf area per leaf dry mass) were converted to LMA (leaf dry mass per leaf area): LMA = 1/SLA.

#### - Imputation of N_mass_

Trait records for leaf nitrogen content per leaf area (N_area_) were converted to records of leaf nitrogen content per leaf dry mass (N_mass_) if records for LMA were available for the same observation (leaf): N_mass_ = N_area_/LMA.

#### - Imputation of SM

To be able to include trait data for pteridophytes in the analyses in Díaz *et al*.^9^, diaspore mass values were estimated based on published data for spore radius (r). We assumed that spores would be approximately spherical, with volume = (4/3)πr^3^, and that their density would be 0.5 mg mm^−3^ (refs. ^237–240^). Although these assumptions were imprecise, we are confident they result in spore masses within the right order of magnitude and several orders of magnitude smaller than seed mass of spermatophytes. Most data were from Page^237^, data for *Sadleria pallida* were from Lloyd^238^, for *Pteridium aquilinum* from Conway^239^, and for *Diphasiastrum* spp from Stoor *et al*.^240^.

#### Probabilistic outlier detection

The hierarchical taxonomic classification of plants into families, genera and species has been shown to be highly informative with respect to the probability of trait values^241–243^. We therefore used it to conduct outlier detection at each of these levels.

The six core traits provided in the Global Spectrum Dataset are approximately normally distributed on a logarithmic scale^10^. We therefore assume that on log-scale, traits sample from normal distributions. In the context of a normal distribution the density distribution is symmetric to the mean with 99.73% (99.99%) of data to be expected within the range of mean +/− 3 standard deviations, and 99.99% of data within +/− 4 standard deviations. Using these wide confidence intervals ensures that extreme values that correspond to truly extreme values of traits in nature are not mistakenly identified as outliers and therefore excluded from the dataset.

The z-score indicates how many standard deviations a record is away from the mean:

$${\rm{z}} \mbox{-} {\rm{s}}{\rm{c}}{\rm{o}}{\rm{r}}{\rm{e}}=({\rm{v}}{\rm{a}}{\rm{l}}{\rm{u}}{\rm{e}}-{\rm{m}}{\rm{e}}{\rm{a}}{\rm{n}})/{\rm{s}}{\rm{t}}{\rm{a}}{\rm{n}}{\rm{d}}{\rm{a}}{\rm{r}}{\rm{d}}\,{\rm{d}}{\rm{e}}{\rm{v}}{\rm{i}}{\rm{a}}{\rm{t}}{\rm{i}}{\rm{o}}{\rm{n}}$$

Trait values with absolute z-scores >4 (>3) have a probability of less than 0.1% (0.3%) to be true values of the normal distribution. These trait values are most probably caused by errors not yet detected for these individual records, e.g., wrong unit, decimal error of trait value, wrong species (e.g. by mistake attributing a herb species name to a height measured on a tree), problems related to the trait definition or non-representative growth or measurement conditions. We acknowledge however that our z-score cutoff choice is an arbitrary one.

**Fig. 1:**
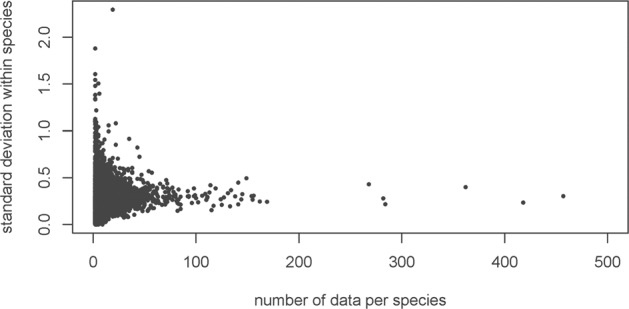
Scatterplot indicating the relation of standard deviation within species and sample size on the example of SLA data (1/LMA) derived from the TRY database version 1 (Kattge *et al*.^10^, Fig. S1).

In many cases the number of trait values per taxon (e.g. a given species) was too small for a representative sample and did not provide a reliable estimate of the standard deviation (see Fig. 1). To circumvent this problem, we used the average standard deviation of trait values at the given taxonomic level, e.g., species, genus, family or all vascular plants. This average is an approximation of the standard deviation to be expected for an individual taxon, if a sufficient number of observations would be available (Fig. 1)^10^.

This probability-based data quality assessment on the different levels of the taxonomic hierarchy is routinely conducted within the TRY database for all traits with more than 1000 records. The z-score values for each trait record are made available on the TRY website and the highest absolute value is provided with each data release.

Trait values with an absolute z-score >4 (more than 4 standard deviations from at least one taxon mean) were excluded from the dataset unless their retention could be justified from external sources. Trait records with an absolute z-score 3 to 4 (3 to 4 standard deviations from at least one taxon mean) were checked by domain experts among the authors for plausibility, and retained or excluded accordingly.

#### Exclusion of duplicate trait records

Duplicate trait records were identified on the basis of the following criteria: same species (after standardization of taxonomy), similar trait values (accounting for rounding errors after semantic integration, unit conversion and data complementation), and no information on different measurement locations or dates.

#### Calculation of species mean trait values

The resulting dataset was used to calculate species mean trait values, without further stratification along, e.g., datasets or measurement sites. As trait distributions of the six core traits have been shown to be log-normal^9^, the mean species trait values were calculated after log-transformation of the trait values (geometric mean).

#### Addition of categorical traits

Data for the categorical traits were added and, if in doubt, checked against expert knowledge and independent external information from specialized websites in the Internet.

#### Final validation of taxonomy and mean trait values

**Fig. 2:**
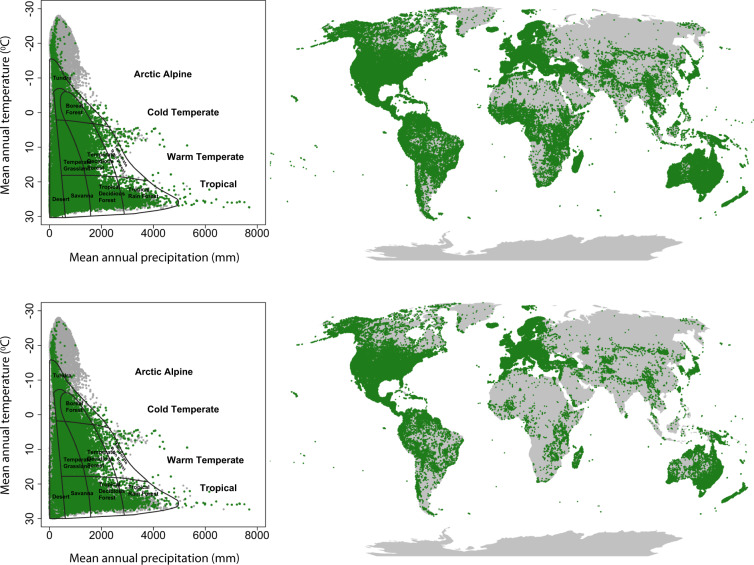
Climatic and geographical coverage of the dataset. Green points, occurrences according to the Global Biodiversity Information Facility (GBIF) (http://www.gbif.org) of species with information on at least one core trait (upper panels) and all six core traits (lower panels). Right panels show distribution in the global map (Robinson projection); grey: land surface. Maps are based on the R package ‘maps’, accessed at The Comprehensive R Archive Network (https://cran.r-project.org/web/packages/maps/index.html). Left panels show distribution in major climatic regions of the world; grey: MAP and MAT as in Climate Research Unit (CRU) CL v.1.0 0.5 degree climatology (http://www.cru.uea.ac.uk/data, ref. ^245^); Biome classification according to Whittaker^246^. This figure is reproduced from ref. ^9^ with permission.

Taxonomy was finally checked once more manually against the Plant List and APGIII. The ten most extreme species mean values of each trait (smallest and largest) were checked manually for reliability against external sources. Finally, outliers of species mean traits – after categorization of species according to the categorical traits and in bi- and multivariate trait space – were validated against external sources (see Díaz *et al*.^9^ Fig. 2, Extended Data Fig. 3, and Extended Data Fig. 4).

## Data Records

The dataset is available under a CC-BY license at the TRY File Archive (https://www.try-db.org/TryWeb/Data.php):

Díaz, S. *et al*. The global spectrum of plant form and function: enhanced species-level trait dataset. TRY File Archive 10.17871/TRY.81 (2022)^244^

### The dataset consists of two data files

- Species_mean_traits.xlsx
- References.xlsx

### Species_mean_traits.xlsx

The file provides mean trait values of plants grown under natural conditions for 46,047 species (including a small number of genus level classifications, sub-species and local varieties). Species names and mean trait values are complemented by taxonomic hierarchy (genus, family and phylogenetic group), the number of trait records contributing to each mean trait value and by categorical traits. Values of all six traits were available for 2,214 species. In total the dataset contains 476,932 entries for quantitative and categorical trait records and higher-level taxonomy (92,159 entries for quantitative traits, 200,585 entries for categorical traits, and 184,188 entries for higher-level taxonomy).

The quantitative species-level trait information is based on about 1 million trait records (see Table S1), measured on >500,000 plant individuals (number of different Observations in References (see below)). One trait record reported in the datasets is often based on several replicated measurements from different representative individuals at a site. The New Handbook for Standardised Measurement of Plant Functional Traits Worldwide^6^ recommends measurements on 10 to 25 individual plants or leaves, depending on the trait. Therefore in the cases that followed this or related protocols, a trait record in the original database probably represents the site-specific mean trait value for a given species. Reporting only the site-specific mean trait value was standard procedure in older publications and aggregated databases, assuming a common approach to replicated measurements on different individuals. More recent datasets tend to provide all individual measurements, among other reasons because this allows better treatment of intraspecific trait variation.

The present dataset was derived from 157 datasets (Table S1). Trait records can be traced to ca. 2500 original publications (see References_original_sources.xlsx). All species are complemented with higher-level taxonomic information; 92.5% and 84.8% of species are attributed to categories according to woodiness and basic growth-form, respectively. The raw data are available via the TRY Database (https://www.try-db.org/TryWeb/Home.php).

### References.xlsx

This file contains the references of all trait data, which contributed to the core traits of the Global Spectrum Dataset via the TRY database. If datasets contributed to TRY were already compiled from original publications, the table also provides the references of these original publications. The references are linked to the data in the species mean trait dataset via species unique identifiers and trait names.

The sum of replicates in the species mean trait table is about 100,000 trait records less than the sum of 979,924 trait records in References and Supplementary Table S1, because the species mean trait table contains mean trait values and information on number of trait records only for those species-trait combinations that were retained after data cleaning and imputation.

## Technical Validation

**Fig. 3:**
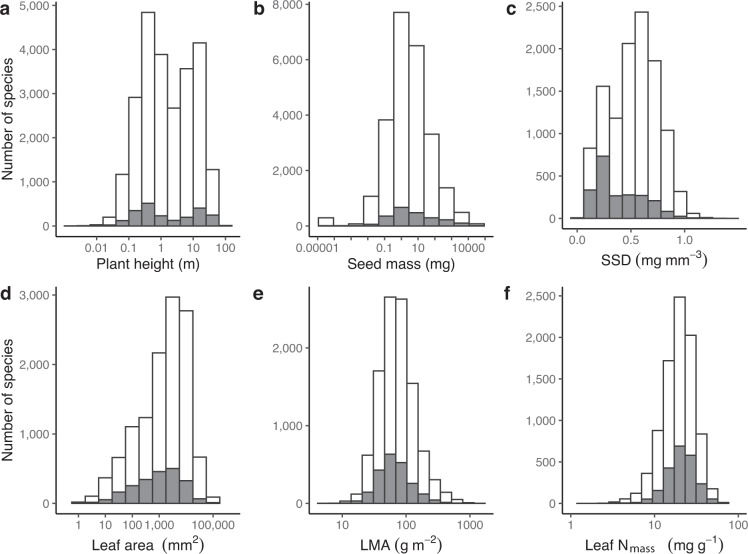
Frequency distributions of species for the six core traits. Grey: species with all six traits; white: species with at least one trait. (**a**) Plant height, (**b**) Seed mass, (**c**) SSD: stem dry mass per stem fresh volume (stem specific density), (**d**) Leaf area, (**e**) LMA: leaf dry mass per leaf area, (**f**) Leaf N_mass_: leaf nitrogen content per leaf dry mass (leaf nitrogen concentration).

**Fig. 4:**
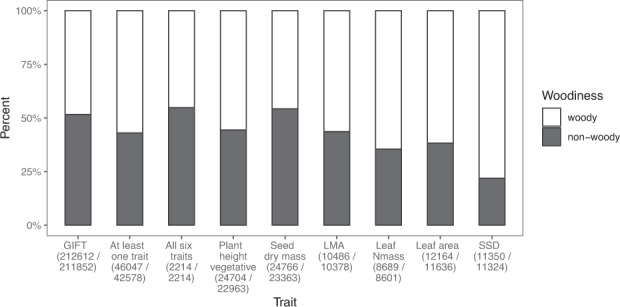
The coverage of species per trait with respect to woodiness (woody versus non-woody incl. semi-woody). The coverage in the GIFT database^247^^,^^248^ a comprehensive baseline of plant growth form, is included for external comparison (see ref. ^11^ for more details). In parentheses: the number of species with data for the trait and the number of species for which woodiness could be determined.

The dataset has a global coverage in geographic and climate space (Fig. 2, also Díaz *et al*.^9^ Extended Data Fig. 1), however with known gaps^9–11^. The numbers of species characterized per trait are similar to the TRY Database version 5, published in 2019^11^. This indicates the efficiency of data collection and curation for the Global Spectrum Dataset. All species mean trait values (Table 2) are within the ranges published in Kattge *et al*.^10^. Histograms of trait frequency distributions are provided in Fig. 3. The coverage of species per trait with respect to woodiness is presented in Fig. 4. The dataset has so far been used in Díaz *et al*.^9^, where the data show a high internal consistency in bi- and multivariate analyses: known bivariate relationships were well reproduced (Díaz *et al*.^9^ Extended Data Figs. 3 and 4) and individual species were located in the first axes of the principal component analysis in positions expected from general knowledge about these species (Díaz *et al*.^9^ Fig. 2).

**Table 2:** Number of species and range of variation of species mean traits, geographic distributions and climatic conditions in the Global Spectrum Dataset.

	No. of species	Mean	Range			Mean & median n per species
			Min.		Max.	
H	24704	1.62	0.001	*to*	90	9.5 (2)
SSD	11350	0.47	0.06	*to*	1.39	8.9 (2)
LA	12164	1336	0.8	*to*	2.79E6	10 (3)
LMA	10486	72.4	4.9	*to*	1507	15.4 (5)
N_mass_	8689	19.2	2.48	*to*	68.98	9.3 (3)
SM	24766	2.65	5.15E-6	*to*	2.05E7	8.6 (6)
Latitude			55 S	*to*	83.17 N	
Altitude			−59	*to*	5249	
MAT			−27.22	*to*	29.97	
MAP			<5	*to*	7693	

No. of species: number of species characterized; Mean: geometric mean of species traits; Range: lowest and highest species mean trait values; Mean & median n per species: mean and median (in brackets) number of trait records per species; H: adult plant height (m); SSD: stem specific density (mg mm^−3^); LA: leaf area (mm^2^); LMA: leaf mass per area (g m^−2^); N_mass_: N content per unit leaf mass (mg g^−1^); SM: diaspore (seed or spore) mass (mg); Latitude in degrees; Altitude in m; MAT: Mean annual temperature (°C); MAP: Mean annual precipitation (mm). Mean annual temperature and precipitation refer to CRU0.5 degree climatology. Modified from ref. ^9^ with permission.

## Usage Notes

In case the dataset is used in publications, both this paper and Díaz *et al*.^9^ should be cited.

The six quantitative traits compiled here (plus LDMC) are among the best-covered quantitative traits in the TRY database. However, as is typical for these kinds of observational data, the numbers of records per species are unevenly distributed: few species mean trait values are based on a large number of records, while a large fraction of the species mean estimates is based on only a few or a single trait record(s) (see difference between mean and median number of trait records per species and trait in Table 2, the number of trait records per species mean is also indicated in the dataset file ‘Species_mean_traits.xlsx’). The representativeness of these mean values should be taken with caution, because the trait measurements have to be treated as samples from the variation of traits within species, which – for some traits – can be substantial^10^. However, as mentioned above, one trait record is often based on several trait measurements on characteristic individuals and therefore represents a species per site-specific mean value. In the context of large-scale analyses the variation within species has been shown to be considerably smaller than the variation between species^10^.

## Supplementary information

Supplementary Table S1

## Data Availability

Does not apply.
