# Supplementary material for: The global spectrum of plant form and function: enhanced species-level trait dataset
Source: Sci Data. 2022 Dec 7;9:755. doi: 10.1038/s41597-022-01774-9 (PMC9729214; doi:10.1038/s41597-022-01774-9)
Supplement: Supplementary file 1 — Supplementary Table S1 [file 41597_2022_1774_MOESM1_ESM.docx]

**Supplementary table to Díaz et al. ‘The global spectrum of plant form and function: enhanced species-level trait dataset’ Scientific Data.**

**Supplementary Table 1:** Contributing datasets (sorted by custodian last name). Citation numbers relate to the reference list and to the main publication. Institution relates to the affiliations under which the individual datasets were collected and/or contributed to the joint dataset.

| **Dataset** | **Custodian** | **Institution** | **Number of trait records** |
| --- | --- | --- | --- |
| Functional Traits of Graminoids in Semi-Arid Steppes Database^17,18^ | Peter Adler | Utah State University | 174 |
| French Weeds Trait Database (unpublished) | Bernard Amiaud | Nancy Université - INRA | 2300 |
| Plant Physiology Database^19-22^ | Owen Atkin | Australian National University | 564 |
| European Mountain Meadows Plant Traits Database^23,24^ | Michael Bahn | University of Innsbruck | 1086 |
| Leaf Photosynthesis and Nitrogen at Oak Rich Dataset^25^ | Dennis Baldocchi | University of California | 134 |
| Photosynthesis Traits Database^26^ | Dennis Baldocchi | University of California | 936 |
| The Bridge Database^27,28^ | Christopher Baraloto | Florida International University | 45380 |
| Leaf Structure, Venation and Economic Spectrum^29-32^ | Benjamin Blonder | University of California at Berkeley | 10353 |
| Photosynthesis and Leaf Characteristics Database (unpublished) | Benjamin Blonder | University of California at Berkeley | 976 |
| South African Woody Plants Database (ZLTP) (unpublished) | William Bond | University of Cape Town | 854 |
| Plant Traits of Canadian Forests^33-35^ | Benjamin Bond-Lamberty | University of Maryland–College Park | 3611 |
| Leaf Traits From Madagascar^36^ | Kerry Brown | Kingston University London | 1362 |
| Plant Traits from Circeo National Park, Italy^37^ | Sabina Burrascano | University of Rome La Sapienza | 1270 |
| Traits of US Desert Woody Plant Species^38^ | Bradley Butterfield | University of California | 2223 |
| SLA and LDMC for Canadian Wetland Species^39^ | Chaeho Byun | Andong National University | 775 |
| Leaf Traits in Central Apennines Beech Forests^40^ | Giandiego Campetella | University of Camerino | 7074 |
| Floridian Leaf Traits Database^41^ | Jeannine Cavender-Bares | University of Minnesota | 7809 |
| Flora d’Italia Functional Traits Hoard (FIFTH)^42^ | Bruno Cerabolini | University of Insubria | 15219 |
| Hydrophytes Traits Database^43^ | Bruno Cerabolini | University of Insubria | 1815 |
| Tundra Plant Traits Database (unpublished) | F. Stuart III Chapin | University of Alaska Fairbanks | 146 |
| Abisko & Sheffield Database^44-47^ | Johannes Cornelissen | Vrije Universiteit Amsterdam | 1433 |
| Sheffield & Spain Woody Database^48-50^ | Johannes Cornelissen | Vrije Universiteit Amsterdam | 1008 |
| Sheffield Database^46,48,51,52^ | Johannes Cornelissen | Vrije Universiteit Amsterdam | 10268 |
| ArtDeco Database^53^ | Will Cornwell | UNSW Sydney | 1096 |
| Jasper Ridge Californian Woody Plants Database^54-57^ | Will Cornwell | UNSW Sydney | 1260 |
| Global 15N Database^58^ | Joseph Craine | Jonah Ventures | 7387 |
| Plant Traits for Grassland Species (Konza Prairie, Kansas, USA)^59-61^ | Joseph Craine | Jonah Ventures | 1276 |
| Roots Of the World (ROW) Database^62^ | Joseph Craine | Jonah Ventures | 218 |
| Panama Tree Traits^63,64^ | Dylan Craven | Universidad Mayor Santiago | 378 |
| Italian Alps Plant Traits Database^65,66^ | Matteo Dainese | Eurac Research | 16116 |
| LBA-ECO CD-02 C and N Isotopes in Leaves, Amazonas, Brazil^67^ | Alessandro de Araujo | EMBRAPA | 32 |
| Leaf N-Retention Database (unpublished) | Franciska de Vries | University of Amsterdam | 234 |
| CORDOBASE^52^ | Sandra Díaz | Universidad Nacional de Córdoba-CONICET | 2511 |
| Sheffield-Iran-Spain Database^52^ | Sandra Díaz | Universidad Nacional de Córdoba-CONICET | 1590 |
| Seed Information Database (SID)^68^ | John Dickie | Royal Botanic Gardens, Kew | 143777 |
| Traits for Herbaceous Species from Andorra (unpublished) | Marta Domènech | Institut d'Estudis Andorrans | 359 |
| LBA ECO Tapajos: Leaf Characteristics and Photosynthesis^69,70^ | Tomas Domingues | Universidade de São Paulo | 2050 |
| TROBIT West Africa^71^ | Tomas Domingues | Universidade de São Paulo | 1088 |
| The Americas N&P database^72^ | Brian Enquist | University of Arizona | 851 |
| Seed Characteristics of Ericaceae^73^ | Jaime Fagundez | University of A Coruña | 16 |
| Chinese Leaf Traits Database^74-76^ | Jingyun Fang | University of Peking | 4044 |
| Traits of Species of Bajo Calima Region, Buenaventura Colombia^77^ | Fernando Fernández-Méndez | Universidad del Tolima | 50 |
| Costa Rica Rainforest Trees Database (unpublished) | Bryan Finegan | Tropical Agricultural Centre for Research and Higher Education (CATIE) | 558 |
| Ecological Flora of the British Isles^78^ | Alastair Fitter | University of York | 8407 |
| Leaf Characteristics of Pinus sylvestris and Picea abies (unpublished) | Katrin Fleischer | VU Amsterdam | 236 |
| Plant Coastal Dune Traits (France, Aquitaine) (unpublished) | Estelle Forey | Normandie University | 216 |
| Plant Functional Traits of Arid Steppes in Eastern Morocco^79^ | Cedric Frenette-Dussault | Géopole de l'Université de Sherbrooke | 13585 |
| Leaf Traits Mount Hutt, New Zealand^80^ | Gregoire Freschet | Paul Sabatier University, Moulis | 120 |
| Traits from Subarctic Plant Species Database^81^ | Gregoire Freschet | Paul Sabatier University, Moulis | 304 |
| BASECO: a floristic and ecological database of Mediterranean French flora^82^ | Sophie Gachet | Aix Marseille Université / Institut Méditerranéen de Biodiversité et d'Ecologie | 2262 |
| Climbing plants trait dataset^83^ | Rachael Gallagher | Macquarie University | 1523 |
| The VISTA Plant Trait Database ^84-87^ | Eric Garnier | Centre National de la Recherche Scientifique, CNRS | 32644 |
| VegClass CBM Global Database^88^ | Andy Gillison | Center for Biodiversity Management | 13599 |
| PLANTATT - Attributes of British and Irish Plants^89^ | Marco Girardello | Centre for Ecology and Hydrology | 1801 |
| Functional Traits for Restoration Ecology in the Colombian Amazon (unpublished) | Andres Gonzalez-Melo | Universidad National de Colombia | 185 |
| PLANTSdata USDA^90^ | Walton Green | Harvard University | 4539 |
| Leaf Gross Morphometrics Within one Species in Relation to Latitude, Altitude and Time^91^ | Greg Guerin | University of Adelaide | 769 |
| VirtualForests Trait Database^92^ | Alvaro G. Gutiérrez | Universidad de Chile | 302 |
| Leaf Ash Content in China's Terrestrial Plants^93^ | Wenxuan Han | China Agricultural University | 926 |
| Leaf Nitrogen and Phosphorus for China's Terrestrial Plants^94^ | Wenxuan Han | China Agricultural University | 497 |
| Chinese Traits^95,96^ | Sandy Harrison | University of Reading | 9488 |
| Harze Trait Intravar: SLA, LDMC and Plant Height for Calcareous Grassland Species in South Belgium (unpublished) | Mélanie Harzé | University of Liège | 1275 |
| Komati Leaf Trait Data (unpublished) | Wesley Hattingh | Nova Pioneer, Global Systems and Analytics | 60 |
| Cold Tolerance, Seed Size and Height of North American Forest Tree Species (unpublished) | Bradford Hawkins | University of California, Irvine | 778 |
| Fire Related Traits in Proteaceae and Pinaceae^97,98^ | Tianhua He | Curtin University | 142 |
| Herbaceous Traits from the Öland Island Database^99^ | Thomas Hickler | Senckenberg Biodiversity and Climate Research Centre | 192 |
| Dispersal Traits Database (unpublished) | Steve Higgins | University of Bayreuth | 133 |
| LABDENDRO Brazilian Subtropical Forest Traits Database (unpublished) | Pedro Higuchi | Santa Catarina State University | 380 |
| Nutrient Resorption Efficiency Database^100,101^ | Robert Jackson | Stanford University | 4400 |
| Growth and Herbivory of Juvenile Trees (unpublished) | Hervé Jactel | Institut National de la Recherche Agronomique (INRA) | 28999 |
| Xylem Functional Traits (XFT) Database^102^ | Steven Jansen | Ulm University | 2042 |
| Leaf Physiology Database^103^ | Jens Kattge | Max Planck Institute for Biogeochemistry | 2754 |
| KEW African Plant Traits Database^104^ | Don Kirkup | Royal Botanic Gardens KEW | 9252 |
| The LEDA Traitbase^16^ | Michael Kleyer | University of Oldenburg | 79009 |
| Plant Trait Database in East and South-East Asia^105,106^ | Fumito Koike | Yokohama National University | 342 |
| Yasuni Ecuador Leaves^107,108^ | Nathan Kraft | University of California Los Angeles (UCLA) | 4542 |
| Baccara - Plant Traits of European Forests (unpublished) | Koen Kramer | Wageningen University | 302 |
| BiolFlor Database^109,110^ | Ingolf Kühn | Helmholtz Centre for Environmental Research – UFZ | 6130 |
| Traits of Bornean Trees Database^111^ | Hiroko Kurokawa | Forestry and Forest Products Research Institute, Tsukuba | 1168 |
| Saskatchewan Plant Trait Database^112,113^ | Eric Lamb | University of Saskatchewan | 360 |
| Meadow Plant Traits: Biomass Allocation, Rooting depth (unpublished) | Vojtech Lanta | University of Turku | 37 |
| Plant Traits for Pinus and Juniperus Forests in Arizona^114,115^ | Daniel Laughlin | University of Wyoming | 9809 |
| New South Wales Plant Traits Database (unpublished) | Michelle Leishman | Macquarie University | 1043 |
| Crown Architecture Database (unpublished) | Felipe Lenti | Universidade de Brasília | 156 |
| The RAINFOR Plant Trait Database^116-118^ | Jon Lloyd | James Cook University | 11847 |
| French Massif Central Grassland Trait Database^119^ | Frédérique Louault | Université Clermont Auvergne, INRAE | 342 |
| Plant Traits from Spanish Mediterranean Shrublands (unpublished) | Fernando Maestre | Universidad Rey Juan Carlos | 5178 |
| Rainfor Leaf Shape, Driptip, Compoundness and Size Database^120^ | Ana Malhado | Universidade Federal de Alagoas | 2425 |
| Reproductive Allocation^121^ | Peter Manning | Senckenberg Biodiversity and Climate Research Centre | 666 |
| The DIRECT Plant Trait Database^122,123^ | Peter Manning | Senckenberg Biodiversity and Climate Research Centre | 435 |
| ECOCRAFT^124-126^ | Belinda Medlyn | Western Sydney University | 2694 |
| Photosynthetic Capacity Dataset^127-129^ | Patrick Meir | Australian National University | 255 |
| Whole Plant Hydraulic Conductance^130^ | Maurizio Mencuccini | CREAF | 29 |
| Panama Leaf Traits Database^131^ | Julie Messier | University of Waterloo | 3784 |
| Altitudinal Vicariants Spain^132^ | Ruben Milla | Universidad Rey Juan Carlos | 2185 |
| Traits of Halohytic Species in North-West-Germany^133,134^ | Vanessa Minden | Vrije Universiteit Brussel | 539 |
| Global Seed Mass, Plant Height Database^135,136^ | Angela Moles | University of New South Wales | 36588 |
| Phalaris arundinacea Genotypes^137,138^ | Jane Molofsky | University of Vermont | 820 |
| Hawaiian Lobeliad^139^ | Rebecca Montgomery | University of Minnesota | 22 |
| Traits from the Wildfire Project^140^ | Marco Moretti | Swiss Federal Research Institute WSL | 74 |
| Global Leaf Robustness and Physiology Database^141,142^ | Ülo Niinemets | Estonian University of Life Sciences | 2350 |
| Plant Traits from Romania^143,144^ | Kinga Öllerer | Institute of Biology, Romanian Academy | 884 |
| Leaf Biomechanics Database^145^ | Yusuke Onoda | Kyoto University | 2297 |
| The Netherlands Plant Traits Database^146,147^ | Jenny Ordonez | Wageningen University | 2572 |
| The Netherlands Plant Height Database (unpublished) | Wim Ozinga | Wageningen Environmental Research | 1999 |
| Impatiens glandulifera Dataset^148^ | Anna Pahl | Technische Universität München | 1065 |
| BROT Plant Trait Database^149,150^ | Juli Pausas | Centro de Investigaciones sobre Desertificación, Valencia | 2140 |
| Plant Traits of Acidic Grasslands in Central Spain^151^ | Begoña Peco | Universidad Autónoma de Madrid | 316 |
| Catalonian Mediterranean Forest Trait Database^152-157^ | Josep Peñuelas | CREAF, Universitat Autònoma de Barcelona | 47496 |
| Catalonian Mediterranean Shrubland Trait Database (unpublished) | Josep Peñuelas | CREAF, Universitat Autònoma de Barcelona | 8 |
| Hawaiian Leaf Traits Database^158,159^ | Josep Peñuelas | CREAF, Universitat Autònoma de Barcelona | 328 |
| Leaf Structure and Economic Spectrum^160-162^ | Simon Pierce | Università di Milano | 6564 |
| ECOQUA South American Plant Traits Database^163-168^ | Valerio Pillar | Universidade Federal do Rio Grande do Sul | 3714 |
| Traits for Herbaceous Species from Andorra (unpublished) | Clara Pladevall | Institut d'Estudis Andorrans | 512 |
| The Tansley Review LMA Database^169^ | Hendrik Poorter | Forschungszentrum Jülich | 3279 |
| Costa Rican Tropical Dry Forest Trees^170^ | Jennifer Powers | University of Minnesota | 320 |
| Leaf Allometry Dataset^171,172^ | Charles Price | University of Western Australia | 7208 |
| Maxfield Meadow, Rocky Mountain Biological Laboratory - LMA (unpublished) | Quentin Read | University of Tennessee | 79 |
| Cedar Creek Savanna SLA, C, N Database^173^ | Peter Reich | University of Minnesota | 2157 |
| Global A, N, P, SLA Database^174^ | Peter Reich | University of Minnesota | 999 |
| Global Respiration Database^175^ | Peter Reich | University of Minnesota | 1633 |
| Reich-Oleksyn Global Leaf N, P Database^174^ | Peter Reich | University of Minnesota | 8475 |
| Herbaceous Plants Traits From Southern Germany (unpublished) | Christine Römermann | Friedrich-Schiller University Jena | 147 |
| Leaf and Whole-Plant Traits Database^176-190^ | Lawren Sack | University of California | 302 |
| California Coastal Grassland Database^191^ | Brody Sandel | Santa Clara University | 3418 |
| Leaf Area, Dry Mass and SLA Dataset (unpublished) | Brandon Schamp | Algoma University | 1628 |
| BIOTREE Trait Data^192^ | Michael Scherer-Lorenzen | University of Freiburg | 216 |
| BIOTREE Trait Shade Experiment^192^ | Michael Scherer-Lorenzen | University of Freiburg | 189 |
| The Xylem/Phloem Database^193,194^ | Fritz Schweingruber | Swiss Federal Research Institute WSL | 7518 |
| Herbs Water Relations on Soil Moisture Gradients^195^ | Serge Sheremetiev | Komarov Botanical Institute | 44837 |
| Tropical Traits from West Java Database^196^ | Satomi Shiodera | Research Institute for Humanity and Nature (RIHN), Kyoto | 159 |
| Leaf and Whole Plant Traits Database^197-207^ | Bill Shipley | Université de Sherbrooke | 7132 |
| Leaf Structure and Chemistry^208,209^ | Bill Shipley | Université de Sherbrooke | 49582 |
| Herbaceous Leaf Traits Database Old Field New York (unpublished) | Andrew Siefert | Syracuse University | 8540 |
| FAPESP Brazil Rainforest Database^164^ | Ênio Sosinski | Embrapa Clima Temperado | 840 |
| Caucasus Plant Traits Database^210,211^ | Nadejda Soudzilovskaia | Leiden University | 780 |
| Niwot Alpine Plant Traits^212^ | Marko Spasojevic | University of California Riverside | 367 |
| Tropical Plant Traits From Borneo Database^213^ | Emily Swaine | University of Aberdeen | 174 |
| Maximum Height of Chinese Tree Species, from Silva Sinica^214^ | Nathan Swenson | University of Maryland | 4540 |
| Plant Functional Traits From the Province of Almeria, Spain (unpublished) | Alexia Totte | Université Libre de Bruxelles | 458 |
| Traits for Common Grasses and Herbs in Spain (unpublished) | Fernando Valladares | Museo Nacional de Ciencias Naturales CSIC | 56 |
| Ukraine Wetlands Plant Traits Database^215,216^ | Peter van Bodegom | Leiden University | 843 |
| Wetland Dunes Database^217-219^ | Peter van Bodegom | Leiden University | 355 |
| Canopy Traits for Temperate Tree Species Under High N-Deposition^220^ | Kris Verheyen | Ghent University | 77 |
| Plant Traits, Virginia, USA^221^ | Betsy von Holle | National Science Foundation, Alexandria | 97 |
| Midwestern and Southern US Herbaceous Species Trait Database (unpublished) | Evan Weiher | University of Wisconsin - Eau Claire | 10240 |
| LBA-ECO CD-09 Soil and Vegetation Characteristics, Tapajos National Forest, Brazil^222^ | Mathew Williams | University of Edinburgh | 58 |
| The Functional Ecology of Trees (FET) Database - Jena^223^ | Christian Wirth | University of Leipzig | 29942 |
| Fonseca/Wright New South Wales Database^224,225^ | Ian Wright | Macquarie University | 5060 |
| GLOPNET - Global Plant Trait Network Database^226,227^ | Ian Wright | Macquarie University | 16868 |
| Neotropical Plant Traits Database^228^ | Ian Wright | Macquarie University | 5521 |
| Overton/Wright New Zealand Database (unpublished) | Ian Wright | Macquarie University | 3135 |
| Leaf Economic Traits Across Varying Environmental Conditions^229^ | Justin Wright | Duke University | 1520 |
| Panama Plant Traits Database^230^ | S. Joseph Wright | Smithsonian Tropical Research Institute | 27941 |
| *Quercus* Leaf C&N Database^231^ | Benjamin Yguel | Centre National de la Recherche Scientifique CNRS | 767 |
| Global Wood Density Database^232,233^ | Amy Zanne | George Washington University, Washington | 33518 |
| San Lorenzo Epiphyte Leaf Traits Database (unpublished) | Gerhard Zotz | University of Oldenburg | 2452 |
|  |  |  | **Σ = 979924** |

**References**

16 Kleyer, M. *et al.* The LEDA Traitbase: a database of life-history traits of the Northwest European flora. *Journal of Ecology* **96**, 1266-1274, doi:10.1111/j.1365-2745.2008.01430.x (2008).

17 Adler, P. B., Milchunas, D. G., Lauenroth, W. K., Sala, O. E. & Burke, I. C. Functional traits of graminoids in semi-arid steppes: a test of grazing histories. *Journal of Applied Ecology* **41**, 653-663, doi:10.1111/j.0021-8901.2004.00934.x (2004).

18 Adler, P. B. *A comparison of livestock grazing effects on sagebrush steppe, USA, and Patagonian steppe, Argentina.* PhD thesis, Colorado State University, (2003).

19 Atkin, O. K., Westbeek, M. H. M., Cambridge, M. L., Lambers, H. & Pons, T. L. Leaf Respiration in Light and Darkness (A Comparison of Slow- and Fast-Growing Poa Species). *Plant Physiology* **113**, 961-965, doi:10.1104/pp.113.3.961 (1997).

20 Campbell, C. *et al.* Acclimation of photosynthesis and respiration is asynchronous in response to changes in temperature regardless of plant functional group. *New Phytologist* **176**, 375-389, doi:10.1111/j.1469-8137.2007.02183.x (2007).

21 Atkin, O. K., Schortemeyer, M., McFarlane, N. & Evans, J. R. The response of fast- and slow-growing Acacia species to elevated atmospheric CO 2 : an analysis of the underlying components of relative growth rate. *Oecologia* **120**, 544-554, doi:10.1007/s004420050889 (1999).

22 Loveys, B. R. *et al.* Thermal acclimation of leaf and root respiration: an investigation comparing inherently fast- and slow-growing plant species. *Global Change Biology* **9**, 895-910, doi:10.1046/j.1365-2486.2003.00611.x (2003).

23 Bahn, M. *et al.* in *Land-use changes in European mountain ecosystems. ECOMONT- Concept and Results* (eds A. Cernusca, U. Tappeiner, & N. Bayfield) 247-255 (Blackwell Wissenschaft, Berlin, 1999).

24 Wohlfahrt, G. *et al.* Inter-specific variation of the biochemical limitation to photosynthesis and related leaf traits of 30 species from mountain grassland ecosystems under different land use. *Plant, Cell and Environment* **22**, 1281-1296, doi:10.1046/j.1365-3040.1999.00479.x (1999).

25 Wilson, K. B., Baldocchi, D. D. & Hanson, P. J. Spatial and seasonal variability of photosynthetic parameters and their relationship to leaf nitrogen in a deciduous forest. *Tree Physiology* **20**, 565-578, doi:10.1093/treephys/20.9.565 (2000).

26 Xu, L. & Baldocchi, D. D. Seasonal trends in photosynthetic parameters and stomatal conductance of blue oak (Quercus douglasii) under prolonged summer drought and high temperature. *Tree Physiology* **23**, 865-877, doi:10.1093/treephys/23.13.865 (2003).

27 Baraloto, C. *et al.* Decoupled leaf and stem economics in rain forest trees. *Ecology Letters* **13**, 1338-1347, doi:10.1111/j.1461-0248.2010.01517.x (2010).

28 Baraloto, C. *et al.* Functional trait variation and sampling strategies in species-rich plant communities. *Functional Ecology* **24**, 208-216, doi:10.1111/j.1365-2435.2009.01600.x (2010).

29 Blonder, B. *et al.* The leaf-area shrinkage effect can bias paleoclimate and ecology research. *American Journal of Botany* **99**, 1756-1763, doi:10.3732/ajb.1200062 (2012).

30 Blonder, B. *et al.* Testing models for the leaf economics spectrum with leaf and whole-plant traits inArabidopsis thaliana. *AoB Plants* **7**, plv049, doi:10.1093/aobpla/plv049 (2015).

31 Blonder, B., Violle, C. & Enquist, B. J. Assessing the causes and scales of the leaf economics spectrum using venation networks inPopulus tremuloides. *Journal of Ecology* **101**, 981-989, doi:10.1111/1365-2745.12102 (2013).

32 Blonder, B., Violle, C., Bentley, L. P. & Enquist, B. J. Venation networks and the origin of the leaf economics spectrum. *Ecology Letters* **14**, 91-100, doi:10.1111/j.1461-0248.2010.01554.x (2010).

33 Bond-Lamberty, B., Wang, C. & Gower, S. T. Aboveground and belowground biomass and sapwood area allometric equations for six boreal tree species of northern Manitoba. *Canadian Journal of Forest Research* **32**, 1441-1450, doi:10.1139/x02-063 (2002).

34 Bond-Lamberty, B., Wang, C., Gower, S. T. & Norman, J. Leaf area dynamics of a boreal black spruce fire chronosequence. *Tree Physiology* **22**, 993-1001, doi:10.1093/treephys/22.14.993 (2002).

35 Bond-Lamberty, B., Wang, C. & Gower, S. T. The use of multiple measurement techniques to refine estimates of conifer needle geometry. *Canadian Journal of Forest Research* **33**, 101-105, doi:10.1139/x02-166 (2003).

36 Brown, K. A. *et al.* Assessing Natural Resource Use by Forest-Reliant Communities in Madagascar Using Functional Diversity and Functional Redundancy Metrics. *PLoS ONE* **6**, e24107, doi:10.1371/journal.pone.0024107 (2011).

37 Burrascano, S. *et al.* Wild boar rooting intensity determines shifts in understorey composition and functional traits. *Community Ecology* **16**, 244-253, doi:10.1556/168.2015.16.2.12 (2015).

38 Butterfield, B. J. & Briggs, J. M. Regeneration niche differentiates functional strategies of desert woody plant species. *Oecologia* **165**, 477-487, doi:10.1007/s00442-010-1741-y (2010).

39 Byun, C., de Blois, S. & Brisson, J. Plant functional group identity and diversity determine biotic resistance to invasion by an exotic grass. *Journal of Ecology* **101**, 128-139, doi:10.1111/1365-2745.12016 (2012).

40 Campetella, G. *et al.* Patterns of plant trait–environment relationships along a forest succession chronosequence. *Agriculture, Ecosystems & Environment* **145**, 38-48, doi:10.1016/j.agee.2011.06.025 (2011).

41 Cavender-Bares, J., Keen, A. & Miles, B. Phylogenetic structure of floridian plant communities depends on taxonomic and spatial scale. *Ecology* **87**, S109-S122, doi:10.1890/0012-9658(2006)87[109:psofpc]2.0.co;2 (2006).

42 Cerabolini, B. E. L. *et al.* Can CSR classification be generally applied outside Britain? *Plant Ecology* **210**, 253-261, doi:10.1007/s11258-010-9753-6 (2010).

43 Pierce, S., Brusa, G., Sartori, M. & Cerabolini, B. E. L. Combined use of leaf size and economics traits allows direct comparison of hydrophyte and terrestrial herbaceous adaptive strategies. *Annals of Botany* **109**, 1047-1053, doi:10.1093/aob/mcs021 (2012).

44 Cornelissen, J. H. C. *et al.* Leaf digestibility and litter decomposability are related in a wide range of subarctic plant species and types. *Functional Ecology* **18**, 779-786, doi:10.1111/j.0269-8463.2004.00900.x (2004).

45 Quested, H. M. *et al.* Decomposition of sub-arctic plants with differenting nitogen economies: a functional role for hemiparasites. *Ecology* **84**, 3209-3221, doi:10.1890/02-0426 (2003).

46 Cornelissen, J. H. C., Diez, P. C. & Hunt, R. Seedling Growth, Allocation and Leaf Attributes in a Wide Range of Woody Plant Species and Types. *The Journal of Ecology* **84**, 755, doi:10.2307/2261337 (1996).

47 Cornelissen, J. H. C., Werger, M. J. A., CastroDiez, P., vanRheenen, J. W. A. & Rowland, A. P. Foliar nutrients in relation to growth, allocation and leaf traits in seedlings of a wide range of woody plant species and types. *Oecologia* **111**, 460-469 (1997).

48 Cornelissen, J. H. C. *et al.* Functional traits of woody plants: correspondence of species rankings between field adults and laboratory-grown seedlings? *Journal of Vegetation Science* **14**, 311, doi:10.1658/1100-9233(2003)014[0311:ftowpc]2.0.co;2 (2003).

49 Castro-Díez, P., Puyravaud, J. P., Cornelissen, J. H. C. & Villar-Salvador, P. Stem anatomy and relative growth rate in seedlings of a wide range of woody plant species and types. *Oecologia* **116**, 57-66, doi:10.1007/s004420050563 (1998).

50 Cornelissen, J. H. C. A triangular relationship between leaf size and seed size among woody species: allometry, ontogeny, ecology and taxonomy. *Oecologia* **118**, 248-255, doi:10.1007/s004420050725 (1999).

51 Cornelissen, J. H. C. An Experimental Comparison of Leaf Decomposition Rates in a Wide Range of Temperate Plant Species and Types. *The Journal of Ecology* **84**, 573, doi:10.2307/2261479 (1996).

52 Díaz, S. *et al.* The plant traits that drive ecosystems: Evidence from three continents. *Journal of Vegetation Science* **15**, 295-304, doi:10.1111/j.1654-1103.2004.tb02266.x (2004).

53 Cornwell, W. K. *et al.* Plant species traits are the predominant control on litter decomposition rates within biomes worldwide. *Ecology Letters* **11**, 1065-1071, doi:10.1111/j.1461-0248.2008.01219.x (2008).

54 Preston, K. A., Cornwell, W. K. & DeNoyer, J. L. Wood density and vessel traits as distinct correlates of ecological strategy in 51 California coast range angiosperms. *New Phytologist* **170**, 807-818, doi:10.1111/j.1469-8137.2006.01712.x (2006).

55 Cornwell, W. K., Schwilk, D. W. & Ackerly, D. D. A trait-based test for habitat filtering: Convex hull volume. *Ecology* **87**, 1465-1471, doi:10.1890/0012-9658(2006)87[1465:attfhf]2.0.co;2 (2006).

56 Ackerly, D. D. & Cornwell, W. K. A trait-based approach to community assembly: partitioning of species trait values into within- and among-community components. *Ecology Letters* **10**, 135-145, doi:10.1111/j.1461-0248.2006.01006.x (2007).

57 Cornwell, W. K. & Ackerly, D. D. Community assembly and shifts in plant trait distributions across an environmental gradient in coastal California. *Ecological Monographs* **79**, 109-126, doi:10.1890/07-1134.1 (2009).

58 Craine, J. M. *et al.* Global patterns of foliar nitrogen isotopes and their relationships with climate, mycorrhizal fungi, foliar nutrient concentrations, and nitrogen availability. *New Phytologist* **183**, 980-992, doi:10.1111/j.1469-8137.2009.02917.x (2009).

59 Craine, J. M. *et al.* Functional consequences of climate change-induced plant species loss in a tallgrass prairie. *Oecologia* **165**, 1109-1117, doi:10.1007/s00442-011-1938-8 (2011).

60 Craine, J. M., Towne, E. G., Ocheltree, T. W. & Nippert, J. B. Community traitscape of foliar nitrogen isotopes reveals N availability patterns in a tallgrass prairie. *Plant and Soil* **356**, 395-403, doi:10.1007/s11104-012-1141-7 (2012).

61 Tucker, S. S., Craine, J. M. & Nippert, J. B. Physiological drought tolerance and the structuring of tallgrass prairie assemblages. *Ecosphere* **2**, art48, doi:10.1890/es11-00023.1 (2011).

62 Craine, J. M., Lee, W. G., Bond, W. J., Williams, R. J. & Johnson, L. C. Environmental constraints on a global relationship among leaf and root traits of grasses. *Ecology* **86**, 12-19, doi:10.1890/04-1075 (2005).

63 Craven, D. *et al.* Between and within-site comparisons of structural and physiological characteristics and foliar nutrient content of 14 tree species at a wet, fertile site and a dry, infertile site in Panama. *Forest Ecology and Management* **238**, 335-346, doi:10.1016/j.foreco.2006.10.030 (2007).

64 Craven, D. *et al.* Seasonal variability of photosynthetic characteristics influences growth of eight tropical tree species at two sites with contrasting precipitation in Panama. *Forest Ecology and Management* **261**, 1643-1653, doi:10.1016/j.foreco.2010.09.017 (2011).

65 Bragazza, L. Conservation priority of Italian Alpine habitats: a floristic approach based on potential distribution of vascular plant species. *Biodiversity and Conservation* **18**, 2823-2835, doi:10.1007/s10531-009-9609-3 (2009).

66 Dainese, M. & Bragazza, L. Plant traits across different habitats of the Italian Alps: a comparative analysis between native and alien species. *Alpine Botany* **122**, 11-21, doi:10.1007/s00035-012-0101-4 (2012).

67 de Araujo, A. C. *et al.* LBA-ECO CD-02 C and N Isotopes in Leaves and Atmospheric CO_2_, Amazonas, Brazil. Data set. Available on-line [http://daac.ornl.gov ] from Oak Ridge National Laboratory Distributed Active Archive Center, Oak Ridge, Tennessee, U.S.A., (2011).

68 Royal Botanical Gardens KEW. Seed Information Database (SID). Version 7.1. Available from: http://data.kew.org/sid/ (accessed May 2011). (2008).

69 Domingues, T. F., Berry, J. A., Martinelli, L. A., Ometto, J. P. H. B. & Ehleringer, J. R. Parameterization of Canopy Structure and Leaf-Level Gas Exchange for an Eastern Amazonian Tropical Rain Forest (Tapajós National Forest, Pará, Brazil). *Earth Interactions* **9**, 1-23, doi:10.1175/ei149.1 (2005).

70 Domingues, T. F., Martinelli, L. A. & Ehleringer, J. R. Ecophysiological traits of plant functional groups in forest and pasture ecosystems from eastern Amazônia, Brazil. *Plant Ecology* **193**, 101-112, doi:10.1007/s11258-006-9251-z (2007).

71 Domingues, T. F. *et al.* Co-limitation of photosynthetic capacity by nitrogen and phosphorus in West Africa woodlands. *Plant, Cell & Environment* **33**, 959-980, doi:10.1111/j.1365-3040.2010.02119.x (2010).

72 Kerkhoff, A. J., Fagan, W. F., Elser, J. J. & Enquist, B. J. Phylogenetic and Growth Form Variation in the Scaling of Nitrogen and Phosphorus in the Seed Plants. *The American Naturalist* **168**, E103-E122, doi:10.1086/507879 (2006).

73 Fagúndez, J. & Izco, J. Seed morphology of the European species ofEricaL. sect.ArsaceSalisb. ex Benth. (Ericaceae). *Acta Botanica Gallica* **157**, 45-54, doi:10.1080/12538078.2010.10516188 (2010).

74 Han, W., Fang, J., Guo, D. & Zhang, Y. Leaf nitrogen and phosphorus stoichiometry across 753 terrestrial plant species in China. *New Phytologist* **168**, 377-385, doi:10.1111/j.1469-8137.2005.01530.x (2005).

75 He, J.-S. *et al.* A test of the generality of leaf trait relationships on the Tibetan Plateau. *New Phytologist* **170**, 835-848, doi:10.1111/j.1469-8137.2006.01704.x (2006).

76 He, J.-S. *et al.* Leaf nitrogen:phosphorus stoichiometry across Chinese grassland biomes. *Oecologia* **155**, 301-310, doi:10.1007/s00442-007-0912-y (2007).

77 Bocanegra, K., Fernández, F. & Galvis, J. Grupos funcionales de arboles en bosques secundarios de la region Bajo Calima (Buenaventura, Colombia). *Boletín Científico. Centro de Museos. Museo de Historia Natural* **19**, 17-40, doi:10.17151/bccm.2015.19.1.2 (2015).

78 Fitter, A. H. & Peat, H. J. The Ecological Flora Database. *The Journal of Ecology* **82**, 415, doi:10.2307/2261309 (1994).

79 Frenette-Dussault, C., Shipley, B., Léger, J.-F., Meziane, D. & Hingrat, Y. Functional structure of an arid steppe plant community reveals similarities with Grime's C-S-R theory. *Journal of Vegetation Science* **23**, 208-222, doi:10.1111/j.1654-1103.2011.01350.x (2011).

80 Kichenin, E., Wardle, D. A., Peltzer, D. A., Morse, C. W. & Freschet, G. T. Contrasting effects of plant inter- and intraspecific variation on community-level trait measures along an environmental gradient. *Functional Ecology* **27**, 1254-1261, doi:10.1111/1365-2435.12116 (2013).

81 Freschet, G. T., Cornelissen, J. H. C., van Logtestijn, R. S. P. & Aerts, R. Evidence of the ‘plant economics spectrum’ in a subarctic flora. *Journal of Ecology* **98**, 362-373, doi:10.1111/j.1365-2745.2009.01615.x (2010).

82 Freschet, G. T., Cornelissen, J. H. C., van Logtestijn, R. S. P. & Aerts, R. Substantial nutrient resorption from leaves, stems and roots in a subarctic flora: what is the link with other resource economics traits? *New Phytologist* **186**, 879-889, doi:10.1111/j.1469-8137.2010.03228.x (2010).

83 Gallagher, R. V. & Leishman, M. R. A global analysis of trait variation and evolution in climbing plants. *Journal of Biogeography* **39**, 1757-1771, doi:10.1111/j.1365-2699.2012.02773.x (2012).

84 Garnier, E. *et al.* Assessing the Effects of Land-use Change on Plant Traits, Communities and Ecosystem Functioning in Grasslands: A Standardized Methodology and Lessons from an Application to 11 European Sites. *Annals of Botany* **99**, 967-985, doi:10.1093/aob/mcl215 (2007).

85 Pakeman, R. J., Lepš, J., Kleyer, M., Lavorel, S. & Garnier, E. Relative climatic, edaphic and management controls of plant functional trait signatures. *Journal of Vegetation Science* **20**, 148-159, doi:10.1111/j.1654-1103.2009.05548.x (2009).

86 Pakeman, R. J. *et al.* Impact of abundance weighting on the response of seed traits to climate and land use. *Journal of Ecology* **96**, 355-366 (2008).

87 Fortunel, C. *et al.* Leaf traits capture the effects of land use changes and climate on litter decomposability of grasslands across Europe. *Ecology* **90**, 598-611 (2009).

88 Gillison, A. N. & Carpenter, G. A generic plant functional attribute set and grammar for dynamic vegetation description and analysis. *Functional Ecology* **11**, 775-783, doi:10.1046/j.1365-2435.1997.00157.x (1997).

89 Hill, M. O., Preston, C. D. & Roy, D. B. *PLANTATT - attributes of British and Irish Plants: status, size, life history, geography and habitats*. (Huntingdon: Centre for Ecology and Hydrology, 2004).

90 Green, W. USDA PLANTS Compilation, version 1, 09-02-02. (http://bricol.net/downloads/data/PLANTSdatabase/) NRCS: The PLANTS Database (http://plants.usda.gov, 1 Feb 2009). National Plant Data Center: Baton Rouge, LA 70874-74490 USA. (2009).

91 Guerin, G. R., Wen, H. & Lowe, A. J. Leaf morphology shift linked to climate change. *Biology Letters* **8**, 882-886, doi:10.1098/rsbl.2012.0458 (2012).

92 Gutiérrez, A. G. & Huth, A. Successional stages of primary temperate rainforests of Chiloé Island, Chile. *Perspectives in Plant Ecology, Evolution and Systematics* **14**, 243-256, doi:10.1016/j.ppees.2012.01.004 (2012).

93 Han, W. *et al.* Floral, climatic and soil pH controls on leaf ash content in China's terrestrial plants. *Global Ecology and Biogeography* **21**, 376-382, doi:10.1111/j.1466-8238.2011.00677.x (2011).

94 Chen, Y., Han, W., Tang, L., Tang, Z. & Fang, J. Leaf nitrogen and phosphorus concentrations of woody plants differ in responses to climate, soil and plant growth form. *Ecography* **36**, 178-184, doi:10.1111/j.1600-0587.2011.06833.x (2011).

95 Meng, T.-T. *et al.* Responses of leaf traits to climatic gradients: adaptive variation versus compositional shifts. *Biogeosciences* **12**, 5339-5352, doi:10.5194/bg-12-5339-2015 (2015).

96 Prentice, I. C. *et al.* Evidence of a universal scaling relationship for leaf CO2 drawdown along an aridity gradient. *New Phytologist* **190**, 169-180, doi:10.1111/j.1469-8137.2010.03579.x (2010).

97 He, T., Pausas, J. P., Belcher, C. M., Schwilk, D. W. & Lamont, B. B. Fire-adapted traits of Pinus arose in the fiery Cretaceous. *New Phytologist* **194**, 751—759, doi:10.1111/j.1469-8137.2012.04079.x (2012).

98 He, T., Lamont, B. B. & Downs, K. S. Banksias born to burn. *New Phytologist* **191**, 184—196, doi:10.1111/j.1469-8137.2011.03663.x. (2011).

99 Hickler, T. *Plant functional types and community characteristics along environmental gradients on Öland's Great Alvar (Sweden)* Master thesis, University of Lund, Sweden, (1999).

100 Vergutz, L., Manzoni, S., Porporato, A., Novais, R. F. & Jackson, R. B. Global resorption efficiencies and concentrations of carbon and nutrients in leaves of terrestrial plants. *Ecological Monographs* **82**, 205-220, doi:10.1890/11-0416.1 (2012).

101 Vergutz, L., Manzoni, S., Porporato, A., Novais, R. F. & Jackson, R. B. A Global Database of Carbon and Nutrient Concentrations of Green and Senesced Leaves *Oak Ridge National Laboratory Distributed Active Archive Center, Oak Ridge, Tennessee, U.S.A*, doi:10.3334/ORNLDAAC/1106 (2012).

102 Choat, B. *et al.* Global convergence in the vulnerability of forests to drought. *Nature* **491**, 752-755, doi:10.1038/nature11688 (2012).

103 Kattge, J., Knorr, W., Raddatz, T. & Wirth, C. Quantifying photosynthetic capacity and its relationship to leaf nitrogen content for global-scale terrestrial biosphere models. *Global Change Biology* **15**, 976-991, doi:10.1111/j.1365-2486.2008.01744.x (2009).

104 Kirkup, D., Malcolm, P., Christian, G. & Paton, A. Towards a Digital African Flora. *Taxon* **54**, 457, doi:10.2307/25065373 (2005).

105 Koike, F. Plant traits as predictors of woody species dominance in climax forest communities. *Journal of Vegetation Science* **12**, 327-336, doi:10.2307/3236846 (2001).

106 Koike, F., Clout, M., Kawamichi, M., De Poorter, M. & Iwatsuki, K. *Assessment and Control of Biological Invasion Risks*. (Cambridge, UK and Shoukadoh Book Sellers, Kyoto, Japan, and IUCN, Gland, Switzerland, 2006).

107 Kraft, N. J. B. & Ackerly, D. D. Functional trait and phylogenetic tests of community assembly across spatial scales in an Amazonian forest. *Ecological Monographs* **80**, 401-422, doi:10.1890/09-1672.1 (2010).

108 Kraft, N. J. B., Valencia, R. & Ackerly, D. D. Functional Traits and Niche-Based Tree Community Assembly in an Amazonian Forest. *Science* **322**, 580-582, doi:10.1126/science.1160662 (2008).

109 Kühn, I., Durka, W. & Klotz, S. BiolFlor - a new plant-trait database as a tool for plant invasion ecology. *Diversity and Distribution* **10**, 363-365 (2004).

110 Otto, B. Merkmale von Samen, Früchten, generativen Germinulen und generativen Diasporen. In: Klotz, S., Kühn, I. & Durka, W. [eds.]: BIOLFLOR - Eine Datenbank zu biologisch-ökologischen Merkmalen der Gefäßpflanzen in Deutschland. Schriftenreihe für Vegetationskunde 38. Bundesamt für Naturschutz, Bonn. (2002).

111 Kurokawa, H. & Nakashizuka, T. Leaf herbivory and decomposability in a Malaysian tropical rain forest. *Ecology* **89**, 2645-2656, doi:10.1890/07-1352.1 (2008).

112 Guy, A. L., Mischkolz, J. M. & Lamb, E. G. Limited effects of simulated acidic deposition on seedling survivorship and root morphology of endemic plant taxa of the Athabasca Sand Dunes in well-watered greenhouse trials. *Botany* **91**, 176-181, doi:10.1139/cjb-2012-0162 (2013).

113 Mishkolz, J. M. Selecting and evaluating native forage mixtures for the mixed grass prairie. . (University of Saskatchewan, Saskatoon, SK., 2013).

114 Laughlin, D. C., Leppert, J. J., Moore, M. M. & Sieg, C. H. A multi-trait test of the leaf-height-seed plant strategy scheme with 133 species from a pine forest flora. *Functional Ecology* **24**, 493-501, doi:10.1111/j.1365-2435.2009.01672.x (2009).

115 Laughlin, D. C., Fulé, P. Z., Huffman, D. W., Crouse, J. & Laliberté, E. Climatic constraints on trait-based forest assembly. *Journal of Ecology* **99**, 1489-1499, doi:10.1111/j.1365-2745.2011.01885.x (2011).

116 Fyllas, N. M. *et al.* Basin-wide variations in foliar properties of Amazonian forest: phylogeny, soils and climate. *Biogeosciences* **6**, 2677-2708, doi:10.5194/bg-6-2677-2009 (2009).

117 Baker, T. R. *et al.* Do species traits determine patterns of wood production in Amazonian forests? *Biogeosciences* **6**, 297-307, doi:10.5194/bg-6-297-2009 (2009).

118 Patiño, S. *et al.* Branch xylem density variations across the Amazon Basin. *Biogeosciences* **6**, 545-568, doi:10.5194/bg-6-545-2009 (2009).

119 Louault, F., Pillar, V. D., Aufrère, J., Garnier, E. & Soussana, J. F. Plant traits and functional types in response to reduced disturbance in a semi-natural grassland. *Journal of Vegetation Science* **16**, 151-160, doi:10.1111/j.1654-1103.2005.tb02350.x (2005).

120 Malhado, A. C. M. *et al.* Spatial distribution and functional significance of leaf lamina shape in Amazonian forest trees. *Biogeosciences* **6**, 1577-1590, doi:10.5194/bg-6-1577-2009 (2009).

121 Manning, P., Houston, K. & Evans, T. Shifts in seed size across experimental nitrogen enrichment and plant density gradients. *Basic and Applied Ecology* **10**, 300-308, doi:10.1016/j.baae.2008.08.004 (2009).

122 Fry, E. L., Power, S. A. & Manning, P. Trait-based classification and manipulation of plant functional groups for biodiversity-ecosystem function experiments. *Journal of Vegetation Science* **25**, 248-261, doi:10.1111/jvs.12068 (2013).

123 Everwand, G., Fry, E. L., Eggers, T. & Manning, P. Seasonal Variation in the Capacity for Plant Trait Measures to Predict Grassland Carbon and Water Fluxes. *Ecosystems* **17**, 1095-1108, doi:10.1007/s10021-014-9779-z (2014).

124 Medlyn, B. E. & Jarvis, P. G. Design and use of a database of model parameters from elevated [CO2] experiments. *Ecological Modelling* **124**, 69-83, doi:10.1016/s0304-3800(99)00148-9 (1999).

125 Medlyn, B. E. *et al.* Effects of elevated [CO2] on photosynthesis in European forest species: a meta-analysis of model parameters. *Plant, Cell & Environment* **22**, 1475-1495, doi:10.1046/j.1365-3040.1999.00523.x (1999).

126 Medlyn, B. E. *et al.* Stomatal conductance of forest species after long-term exposure to elevated CO2 concentration: a synthesis. *New Phytologist* **149**, 247-264, doi:10.1046/j.1469-8137.2001.00028.x (2001).

127 Meir, P. *et al.* Acclimation of photosynthetic capacity to irradiance in tree canopies in relation to leaf nitrogen concentration and leaf mass per unit area. *Plant, Cell and Environment* **25**, 343-357, doi:10.1046/j.0016-8025.2001.00811.x (2002).

128 Carswell, F. E. *et al.* Photosynthetic capacity in a central Amazonian rain forest. *Tree Physiology* **20**, 179-186, doi:10.1093/treephys/20.3.179 (2000).

129 Meir, P., Levy, P. E., Grace, J. & Jarvis, P. G. Photosynthetic parameters from two contrasting woody vegetation types in West Africa. *Plant Ecology* **192**, 277-287, doi:10.1007/s11258-007-9320-y (2007).

130 Mencuccini, M. The ecological significance of long-distance water transport: short-term regulation, long-term acclimation and the hydraulic costs of stature across plant life forms. *Plant, Cell and Environment* **26**, 163-182, doi:10.1046/j.1365-3040.2003.00991.x (2003).

131 Messier, J., McGill, B. J. & Lechowicz, M. J. How do traits vary across ecological scales? A case for trait-based ecology. *Ecology Letters* **13**, 838-848, doi:10.1111/j.1461-0248.2010.01476.x (2010).

132 Milla, R. & Reich, P. B. Multi-trait interactions, not phylogeny, fine-tune leaf size reduction with increasing altitude. *Annals of Botany* **107**, 455-465, doi:10.1093/aob/mcq261 (2011).

133 Minden, V. & Kleyer, M. Testing the effect-response framework: key response and effect traits determining above-ground biomass of salt marshes. *Journal of Vegetation Science* **22**, 387-401, doi:10.1111/j.1654-1103.2011.01272.x (2011).

134 Minden, V., Andratschke, S., Spalke, J., Timmermann, H. & Kleyer, M. Plant trait–environment relationships in salt marshes: Deviations from predictions by ecological concepts. *Perspectives in Plant Ecology, Evolution and Systematics* **14**, 183-192, doi:10.1016/j.ppees.2012.01.002 (2012).

135 Moles, A. T., Falster, D. S., Leishman, M. R. & Westoby, M. Small-seeded species produce more seeds per square metre of canopy per year, but not per individual per lifetime. *Journal of Ecology* **92**, 384-396, doi:10.1111/j.0022-0477.2004.00880.x (2004).

136 Moles, A. T. *et al.* Factors that shape seed mass evolution. *Proceedings of the National Academy of Sciences* **102**, 10540-10544, doi:10.1073/pnas.0501473102 (2005).

137 Lavergne, S., Muenke, N. J. & Molofsky, J. Genome size reduction can trigger rapid phenotypic evolution in invasive plants. *Annals of Botany* **105**, 109-116, doi:10.1093/aob/mcp271 (2009).

138 Lavergne, S. & Molofsky, J. Increased genetic variation and evolutionary potential drive the success of an invasive grass. *Proceedings of the National Academy of Sciences* **104**, 3883-3888, doi:10.1073/pnas.0607324104 (2007).

139 Givnish, T. J., Montgomery, R. A. & Goldstein, G. Adaptive radiation of photosynthetic physiology in the Hawaiian lobeliads: light regimes, static light responses, and whole-plant compensation points. *American Journal of Botany* **91**, 228-246, doi:10.3732/ajb.91.2.228 (2004).

140 Moretti, M. & Legg, C. Combining plant and animal traits to assess community functional responses to disturbance. *Ecography* **32**, 299-309, doi:10.1111/j.1600-0587.2008.05524.x (2009).

141 Niinemets, U. Global-Scale Climatic Controls of Leaf Dry Mass per Area, Density, and Thickness in Trees and Shrubs. *Ecology* **82**, 453, doi:10.2307/2679872 (2001).

142 Niinemets, Ü. Research review. Components of leaf dry mass per area - thickness and density - alter leaf photosynthetic capacity in reverse directions in woody plants. *New Phytologist* **144**, 35-47, doi:10.1046/j.1469-8137.1999.00466.x (1999).

143 Ciocarlan, V. *The illustrated Flora of Romania. Pteridophyta et Spermatopyta*. 1141 (Editura Ceres, 2009).

144 Sanda, V., Bita-Nicolae, C. D. & Barabas, N. *The flora of spontane and cultivated cormophytes from Romania*. (Editura “Ion Borcea”, Bacau, 2003).

145 Onoda, Y. *et al.* Global patterns of leaf mechanical properties. *Ecology Letters* **14**, 301-312, doi:10.1111/j.1461-0248.2010.01582.x (2011).

146 Ordoñez, J. C. *et al.* Plant Strategies in Relation to Resource Supply in Mesic to Wet Environments: Does Theory Mirror Nature? *The American Naturalist* **175**, 225-239, doi:10.1086/649582 (2010).

147 Ordoñez, J. C. *et al.* Leaf habit and woodiness regulate different leaf economy traits at a given nutrient supply. *Ecology*, 100413130925016, doi:10.1890/09-1509 (2010).

148 Pahl, A. T., Kollmann, J., Mayer, A. & Haider, S. No evidence for local adaptation in an invasive alien plant: field and greenhouse experiments tracing a colonization sequence. *Annals of Botany* **112**, 1921-1930, doi:10.1093/aob/mct246 (2013).

149 Paula, S. *et al.* Fire-related traits for plant species of the Mediterranean Basin. *Ecology* **90**, 1420-1420, doi:10.1890/08-1309.1 (2009).

150 Paula, S. & Pausas, J. G. Burning seeds: germinative response to heat treatments in relation to resprouting ability. *Journal of Ecology* **96**, 543-552, doi:10.1111/j.1365-2745.2008.01359.x (2008).

151 Peco, B., de Pablos, I., Traba, J. & Levassor, C. The effect of grazing abandonment on species composition and functional traits: the case of dehesa grasslands. *Basic and Applied Ecology* **6**, 175-183, doi:10.1016/j.baae.2005.01.002 (2005).

152 Ogaya, R. & Peñuelas, J. Comparative field study of Quercus ilex and Phillyrea latifolia: photosynthetic response to experimental drought conditions. *Environmental and Experimental Botany* **50**, 137-148, doi:10.1016/s0098-8472(03)00019-4 (2003).

153 Ogaya, R. & Penuelas, J. Contrasting foliar responses to drought in Quercus ilex and Phillyrea latifolia. *Biologia Plantarum* **50**, 373-382, doi:10.1007/s10535-006-0052-y (2006).

154 Ogaya, R. & Peñuelas, J. Tree growth, mortality, and above-ground biomass accumulation in a holm oak forest under a five-year experimental field drought. *Plant Ecology* **189**, 291-299, doi:10.1007/s11258-006-9184-6 (2006).

155 Ogaya, R. & Peñuelas, J. Changes in leaf δ13C and δ15N for three Mediterranean tree species in relation to soil water availability. *Acta Oecologica* **34**, 331-338, doi:10.1016/j.actao.2008.06.005 (2008).

156 Sardans, J., Peñuelas, J. & Ogaya, R. Drought-induced changes in C and N stoichiometry in a Quercus ilex Mediterranean forest. *Forest Science* **54**, 513-522 (2008).

157 Sardans, J., Peñuelas, J., Prieto, P. & Estiarte, M. Changes in Ca, Fe, Mg, Mo, Na, and S content in a Mediterranean shrubland under warming and drought. *Journal of Geophysical Research* **113**, doi:10.1029/2008jg000795 (2008).

158 Penñelas, J. *et al.* Faster returns on ‘leaf economics’ and different biogeochemical niche in invasive compared with native plant species. *Global Change Biology* **16**, 2171-2185, doi:10.1111/j.1365-2486.2009.02054.x (2009).

159 Peñuelas, J. *et al.* Higher Allocation to Low Cost Chemical Defenses in Invasive Species of Hawaii. *Journal of Chemical Ecology* **36**, 1255-1270, doi:10.1007/s10886-010-9862-7 (2010).

160 Pierce, S., Brusa, G., Vagge, I. & Cerabolini, B. E. L. Allocating CSR plant functional types: the use of leaf economics and size traits to classify woody and herbaceous vascular plants. *Functional Ecology* **27**, 1002-1010, doi:10.1111/1365-2435.12095 (2013).

161 Pierce, S., Ceriani, R. M., De Andreis, R., Luzzaro, A. & Cerabolini, B. The leaf economics spectrum of Poaceae reflects variation in survival strategies. *Plant Biosystems - An International Journal Dealing with all Aspects of Plant Biology* **141**, 337-343, doi:10.1080/11263500701627695 (2007).

162 Pierce, S., Luzzaro, A., Caccianiga, M., Ceriani, R. M. & Cerabolini, B. Disturbance is the principal α-scale filter determining niche differentiation, coexistence and biodiversity in an alpine community. *Journal of Ecology* **95**, 698-706, doi:10.1111/j.1365-2745.2007.01242.x (2007).

163 Müller, S. C., Overbeck, G. E., Pfadenhauer, J. & Pillar, V. D. Plant Functional Types of Woody Species Related to Fire Disturbance in Forest–Grassland Ecotones. *Plant Ecology* **189**, 1-14, doi:10.1007/s11258-006-9162-z (2006).

164 Pillar, V. D. & Sosinski, E. E. An improved method for searching plant functional types by numerical analysis. *Journal of Vegetation Science* **14**, 323-332, doi:10.1111/j.1654-1103.2003.tb02158.x (2003).

165 Duarte, L. d. S., Carlucci, M. B., Hartz, S. M. & Pillar, V. D. Plant dispersal strategies and the colonization of Araucaria forest patches in a grassland-forest mosaic. *Journal of Vegetation Science* **18**, 847-858, doi:10.1111/j.1654-1103.2007.tb02601.x (2007).

166 Blanco, C., Sosinski, E., Santos, B., Silva, M. & Pillar, V. On the overlap between effect and response plant functional types linked to grazing. *Community Ecology* **8**, 57-65, doi:10.1556/comec.8.2007.1.8 (2007).

167 Overbeck, G. E., Müller, S. C., Pillar, V. D. & Pfadenhauer, J. Fine-scale post-fire dynamics in southern Brazilian subtropical grassland. *Journal of Vegetation Science* **16**, 655, doi:10.1658/1100-9233(2005)016[0655:fpdisb]2.0.co;2 (2005).

168 Overbeck, G. E. & Pfadenhauer, J. Adaptive strategies in burned subtropical grassland in southern Brazil. *Flora - Morphology, Distribution, Functional Ecology of Plants* **202**, 27-49, doi:10.1016/j.flora.2005.11.004 (2007).

169 Poorter, H., Niinemets, Ü., Poorter, L., Wright, I. J. & Villar, R. Causes and consequences of variation in leaf mass per area (LMA): a meta-analysis. *New Phytologist* **182**, 565-588, doi:10.1111/j.1469-8137.2009.02830.x (2009).

170 Powers, J. S. & Tiffin, P. Plant functional type classifications in tropical dry forests in Costa Rica: leaf habit versus taxonomic approaches. *Functional Ecology* **24**, 927-936, doi:10.1111/j.1365-2435.2010.01701.x (2010).

171 Price, C. A. & Enquist, B. J. Scaling of mass and morphology in Dicotyledonous leaves: an extension of the WBE model. *Ecology* **88**, 1132-1141, doi:10.1890/06-1158 (2007).

172 Price, C. A., Enquist, B. J. & Savage, V. M. A general model for allometric covariation in botanical form and function. *Proceedings of the National Academy of Sciences* **104**, 13204-13209, doi:10.1073/pnas.0702242104 (2007).

173 Willis, C. G. *et al.* Phylogenetic community structure in Minnesota oak savanna is influenced by spatial extent and environmental variation. *Ecography*, no-no, doi:10.1111/j.1600-0587.2009.05975.x (2009).

174 Reich, P. B., Oleksyn, J. & Wright, I. J. Leaf phosphorus influences the photosynthesis–nitrogen relation: a cross-biome analysis of 314 species. *Oecologia* **160**, 207-212, doi:10.1007/s00442-009-1291-3 (2009).

175 Reich, P. B. *et al.* Scaling of respiration to nitrogen in leaves, stems and roots of higher land plants. *Ecology Letters* **11**, 793-801, doi:10.1111/j.1461-0248.2008.01185.x (2008).

176 Cavender-Bares, J., Sack, L. & Savage, J. Atmospheric and soil drought reduce nocturnal conductance in live oaks. *Tree Physiology* **27**, 611-620, doi:10.1093/treephys/27.4.611 (2007).

177 Coomes, D. A., Heathcote, S., Godfrey, E. R., Shepherd, J. J. & Sack, L. Scaling of xylem vessels and veins within the leaves of oak species. *Biology Letters* **4**, 302-306, doi:10.1098/rsbl.2008.0094 (2008).

178 Cornwell, W. K., Bhaskar, R., Sack, L., Cordell, S. & Lunch, C. K. Adjustment of structure and function of Hawaiian Metrosideros polymorpha at high vs. low precipitation. *Functional Ecology* **21**, 1063-1071, doi:10.1111/j.1365-2435.2007.01323.x (2007).

179 Dunbar‐Co, S., Sporck, Margaret, J. & Sack, L. Leaf Trait Diversification and Design in Seven Rare Taxa of the Hawaiian Plantago Radiation. *International Journal of Plant Sciences* **170**, 61-75, doi:10.1086/593111 (2009).

180 Hao, G.-Y., Sack, L., Wang, A.-Y., Cao, K.-F. & Goldstein, G. Differentiation of leaf water flux and drought tolerance traits in hemiepiphytic and non-hemiepiphytic Ficus tree species. *Functional Ecology* **24**, 731-740, doi:10.1111/j.1365-2435.2010.01724.x (2010).

181 Hoof, J., Sack, L., Webb, D. T. & Nilsen, E. T. Contrasting Structure and Function of Pubescent and Glabrous Varieties of Hawaiian Metrosideros polymorpha (Myrtaceae) at High Elevation. *Biotropica* **0**, 070606001740001-???, doi:10.1111/j.1744-7429.2007.00325.x (2007).

182 Martin, R. E., Asner, G. P. & Sack, L. Genetic variation in leaf pigment, optical and photosynthetic function among diverse phenotypes of Metrosideros polymorpha grown in a common garden. *Oecologia* **151**, 387-400, doi:10.1007/s00442-006-0604-z (2006).

183 Nakahashi, C. D., Frole, K. & Sack, L. Bacterial Leaf Nodule Symbiosis in Ardisia (Myrsinaceae): Does it Contribute to Seedling Growth Capacity? *Plant Biology* **7**, 495-500, doi:10.1055/s-2005-865853 (2005).

184 Quero, J. L. *et al.* Relating leaf photosynthetic rate to whole-plant growth: drought and shade effects on seedlings of fourQuercusspecies. *Functional Plant Biology* **35**, 725, doi:10.1071/fp08149 (2008).

185 Sack, L. Responses of temperate woody seedlings to shade and drought: do trade-offs limit potential niche differentiation? *Oikos* **107**, 110-127, doi:10.1111/j.0030-1299.2004.13184.x (2004).

186 Sack, L. & Frole, K. Leaf structural diversity is related to hydraulic capacity in tropical rain forest trees. *Ecology* **87**, 483-491, doi:10.1890/05-0710 (2006).

187 Sack, L., Tyree, M. T. & Holbrook, N. M. Leaf hydraulic architecture correlates with regeneration irradiance in tropical rainforest trees. *New Phytologist* **167**, 403-413, doi:10.1111/j.1469-8137.2005.01432.x (2005).

188 Sack, L., Cowan, P. D., Jaikumar, N. & Holbrook, N. M. The 'hydrology' of leaves: co-ordination of structure and function in temperate woody species. *Plant, Cell and Environment* **26**, 1343-1356, doi:10.1046/j.0016-8025.2003.01058.x (2003).

189 Sack, L., Melcher, P. J., Liu, W. H., Middleton, E. & Pardee, T. How strong is intracanopy leaf plasticity in temperate deciduous trees? *American Journal of Botany* **93**, 829-839, doi:10.3732/ajb.93.6.829 (2006).

190 Scoffoni, C., Pou, A., Aasamaa, K. & Sack, L. The rapid light response of leaf hydraulic conductance: new evidence from two experimental methods. *Plant, Cell & Environment* **31**, 1803-1812, doi:10.1111/j.1365-3040.2008.01884.x (2008).

191 Sandel, B., Corbin, J. D. & Krupa, M. Using plant functional traits to guide restoration: a case study in California coastal grassland. *Ecosphere* **2**, doi:10.1890/ES10-00175.1 (2011).

192 Scherer-Lorenzen, M., Schulze, E., Don, A., Schumacher, J. & Weller, E. Exploring the functional significance of forest diversity: A new long-term experiment with temperate tree species (BIOTREE). *Perspectives in Plant Ecology, Evolution and Systematics* **9**, 53-70, doi:10.1016/j.ppees.2007.08.002 (2007).

193 Schweingruber, F. H. & Landolt, W. *The Xylem Database*. (Swiss Federal Research Institute WSL, 2005).

194 Schweingruber, F. H. & Poschlod, P. Growth rings in herbs and shrubs: Life span, age determination and stem anatomy. *Forest, Snow and Landscape Research* **79**, 195-415 (2005).

195 Sheremetev, S. N. *Herbs on the soil moisture gradient (water relations and the structural-functional organization)*. (KMK Scientific Press Ltd, Moscow, 2005).

196 Shiodera, S., Rahajoe, J. S. & Kohyama, T. Variation in longevity and traits of leaves among co-occurring understorey plants in a tropical montane forest. *Journal of Tropical Ecology* **24**, 121-133, doi:10.1017/s0266467407004725 (2008).

197 Shipley, B. Trade-offs between net assimilation rate and specific leaf area in determining relative growth rate: relationship with daily irradiance. *Functional Ecology* **16**, 682-689, doi:10.1046/j.1365-2435.2002.00672.x (2002).

198 Meziane, D. & Shipley, B. Interacting components of interspecific relative growth rate: constancy and change under differing conditions of light and nutrient supply. *Functional Ecology* **13**, 611-622, doi:10.1046/j.1365-2435.1999.00359.x (1999).

199 McKenna, M. F. & Shipley, B. Interacting determinants of interspecific relative growth: Empirical patterns and a theoretical explanation. *Écoscience* **6**, 286-296, doi:10.1080/11956860.1999.11682529 (1999).

200 Shipley, B. & Vu, T.-T. Dry matter content as a measure of dry matter concentration in plants and their parts. *New Phytologist* **153**, 359-364, doi:10.1046/j.0028-646x.2001.00320.x (2002).

201 Shipley, B. & Parent, M. Germination Responses of 64 Wetland Species in Relation to Seed Size, Minimum Time to Reproduction and Seedling Relative Growth Rate. *Functional Ecology* **5**, 111, doi:10.2307/2389561 (1991).

202 Shipley, B. & Lechowicz, M. J. The functional co-ordination of leaf morphology, nitrogen concentration, and gas exchange in40 wetland species. *Écoscience* **7**, 183-194, doi:10.1080/11956860.2000.11682587 (2000).

203 Pyankov, V. I., Kondratchuk, A. V. & Shipley, B. Leaf structure and specific leaf mass: the alpine desert plants of the Eastern Pamirs, Tadjikistan. *New Phytologist* **143**, 131-142, doi:10.1046/j.1469-8137.1999.00435.x (1999).

204 Meziane, D. & Shipley, B. Interacting determinants of specific leaf area in 22 herbaceous species: effects of irradiance and nutrient availability. *Plant, Cell & Environment* **22**, 447-459, doi:10.1046/j.1365-3040.1999.00423.x (1999).

205 Shipley, B. Structured Interspecific Determinants of Specific Leaf Area in 34 Species of Herbaceous Angiosperms. *Functional Ecology* **9**, 312, doi:10.2307/2390579 (1995).

206 Kazakou, E., Vile, D., Shipley, B., Gallet, C. & Garnier, E. Co-variations in litter decomposition, leaf traits and plant growth in species from a Mediterranean old-field succession. *Functional Ecology* **20**, 21-30, doi:10.1111/j.1365-2435.2006.01080.x (2006).

207 Vile, D. *Significations fonctionnelle et ecologique des traits des especes vegetales: exemple dans une succession post-cultural mediterraneenne et generalisations* PhD thesis, Université de Sherbrooke, Sherbrooke (Quebec), (2005).

208 Auger, S. *L'importance de la variabilité interspécifique des traits fonctionnels par rapport à la variabilité intraspécifique chez les jeunes arbres en forêt mature* Msc thesis, Université de Sherbrooke, Sherbrooke (Quebec) (2012).

209 Auger, S. & Shipley, B. Inter-specific and intra-specific trait variation along short environmental gradients in an old-growth temperate forest. *Journal of Vegetation Science* **24**, 419-428, doi:10.1111/j.1654-1103.2012.01473.x (2012).

210 Soudzilovskaia, N. A. *et al.* Functional traits predict relationship between plant abundance dynamic and long-term climate warming. *Proceedings of the National Academy of Sciences* **110**, 18180-18184, doi:10.1073/pnas.1310700110 (2013).

211 Elumeeva, T. G. *et al.* Long-term vegetation dynamic in the Northwestern Caucasus: which communities are more affected by upward shifts of plant species? *Alpine Botany* **123**, 77-85, doi:10.1007/s00035-013-0122-7 (2013).

212 Spasojevic, M. J. & Suding, K. N. Inferring community assembly mechanisms from functional diversity patterns: the importance of multiple assembly processes. *Journal of Ecology* **100**, 652-661, doi:10.1111/j.1365-2745.2011.01945.x (2012).

213 Swaine, E. K. *Ecological and evolutionary drivers of plant community assembly in a Bornean rain forest* PhD thesis, University of Aberdeen, (2007).

214 Zheng, W. *Silva Sinica: Volume 1-4*. (China Forestry Publishing House, Beijing., 1983).

215 Pan, Y., Cieraad, E. & van Bodegom, P. M. Are ecophysiological adaptive traits decoupled from leaf economics traits in wetlands? *Functional Ecology* **33**, 1202-1210, doi:10.1111/1365-2435.13329 (2019).

216 Douma, J. C., Bardin, V., Bartholomeus, R. P. & van Bodegom, P. M. Quantifying the functional responses of vegetation to drought and oxygen stress in temperate ecosystems. *Functional Ecology* **26**, 1355-1365, doi:10.1111/j.1365-2435.2012.02054.x (2012).

217 van Bodegom, P. M., Sorrell, B. K., Oosthoek, A., Bakker, C. & Aerts, R. Separating the effects of partial submergence and soil oxygen demand on plant physiology. *Ecology* **89**, 193-204, doi:10.1890/07-0390.1 (2008).

218 Bakker, C., Van Bodegom, P. M., Nelissen, H. J. M., Ernst, W. H. O. & Aerts, R. Plant responses to rising water tables and nutrient management in calcareous dune slacks. *Plant Ecology* **185**, 19-28, doi:10.1007/s11258-005-9080-5 (2006).

219 Bakker, C., Rodenburg, J. & Van Bodegom, P. M. Effects of Ca- and Fe-rich seepage on P availability and plant performance in calcareous dune soils. *Plant and Soil* **275**, 111-122 (2005).

220 Adriaenssens, S. *Dry deposition and canopy exchange for temperate tree species under high nitrogen deposition* PhD thesis, Ghent University, Ghent, Belgium, (2012).

221 Von Holle, B. & Simberloff, D. Testing Fox's assembly rule: does plant invasion depend on recipient community structure? *Oikos* **105**, 551-563, doi:10.1111/j.0030-1299.2004.12597.x (2004).

222 Williams, M., Shimabokuro, Y. E. & Rastetter, E. B. LBA-ECO CD-09 Soil and Vegetation Characteristics, Tapajos National Forest, Brazil, Dataset. *Oak Ridge National Laboratory Distributed Active Archive Center, Oak Ridge, Tennessee, U.S.A.*, doi:10.3334/ORNLDAAC/1104 (2012).

223 Wirth, C. & Lichstein, J. W. in *Old-Growth Forests* 81-113 (Springer Berlin Heidelberg, 2009).

224 Fonseca, C. R., Overton, J. M., Collins, B. & Westoby, M. Shifts in trait-combinations along rainfall and phosphorus gradients. *Journal of Ecology* **88**, 964-977, doi:10.1046/j.1365-2745.2000.00506.x (2000).

225 McDonald, P. G., Fonseca, C. R., Overton, J. M. & Westoby, M. Leaf-size divergence along rainfall and soil-nutrient gradients: is the method of size reduction common among clades? *Functional Ecology* **17**, 50-57, doi:10.1046/j.1365-2435.2003.00698.x (2003).

226 Wright, I. J. *et al.* The worldwide leaf economics spectrum. *Nature* **428**, 821-827, doi:10.1038/nature02403 (2004).

227 Wright, I. J. *et al.* Irradiance, temperature and rainfall influence leaf dark respiration in woody plants: evidence from comparisons across 20 sites. *New Phytologist* **169**, 309-319, doi:10.1111/j.1469-8137.2005.01590.x (2005).

228 Wright, I. J. *et al.* Relationships Among Ecologically Important Dimensions of Plant Trait Variation in Seven Neotropical Forests. *Annals of Botany* **99**, 1003-1015, doi:10.1093/aob/mcl066 (2006).

229 Wright, J. P. & Sutton-Grier, A. Does the leaf economic spectrum hold within local species pools across varying environmental conditions? *Functional Ecology* **26**, 1390-1398, doi:10.1111/1365-2435.12001 (2012).

230 Wright, S. J. *et al.* Functional traits and the growth-mortality tradeoff in tropical trees. *Ecology*, 100514035422098, doi:10.1890/09-2335 (2010).

231 Yguel, B. *et al.* Phytophagy on phylogenetically isolated trees: why hosts should escape their relatives. *Ecology Letters* **14**, 1117-1124, doi:10.1111/j.1461-0248.2011.01680.x (2011).

232 Zanne, A. E. *et al.* in *Data from: Towards a worldwide wood economics spectrum. Dataset,* https://doi.org/10.5061/dryad.234 (Dryad, 2009 ).

233 Chave, J. *et al.* Towards a worldwide wood economics spectrum. *Ecology Letters* **12**, 351-366, doi:10.1111/j.1461-0248.2009.01285.x (2009).
